# Adding Value to Cassava Genetic Resources Conserved at CIAT—Part II: Fifty Years of Evaluation and Use of Landraces for Variety Development

**DOI:** 10.3390/plants15162462

**Published:** 2026-08-13

**Authors:** Clair H. Hershey, Hernan Ceballos, Sean Fenstemaker, Carlos Iglesias, Nelson Morante, Lizbeth Pino Duran, Peter Wenzl, Jonathan Newby

**Affiliations:** 1 Independent Researcher, Formerly Cassava Program, Centro Internacional de Agricultura Tropical (CIAT), Recta Cali-Palmira, Apartado Aéreo 6713, Cali 763537, Colombia; hernanceballosl54@gmail.com; 2 Cassava Program, Centro Internacional de Agricultura Tropical (CIAT), Recta Cali-Palmira, Apartado Aéreo 6713, Cali 763537, Colombia; s.fenstemaker@cgiar.org (S.F.); n.morante@cgiar.org (N.M.); l.pino@cgiar.org (L.P.D.); 3 Plant Breeding Consortium, North Carolina State University, 2721 Founders Drive, Raleigh, NC 27607, USA; caiglesi@ncsu.edu; 4 Genetic Resources Program, Centro Internacional de Agricultura Tropical (CIAT), Recta Cali-Palmira, Apartado Aéreo 6713, Cali 763537, Colombia; p.wenzl@cgiar.org; 5 Cassava Program, Lao PDR Office, Vientiane P.O. Box 783, Laos; j.newby@cgiar.org

**Keywords:** cassava breeding, cassava landraces, cassava genebank, breeding impact

## Abstract

For millennia, farmers in cassava’s homeland in the neotropics selected varieties suited to their climate, soils, biological environments, management systems, and nutritional needs. These actions were fundamental to the crop’s success as a reliable staple energy source. After the arrival of Europeans in the New World, these varieties spread first to Africa and later to Asia, where they were further selected for local conditions and needs. The founders of the International Center for Tropical Agriculture in Cali, Colombia, recognized the importance of legacy landrace varieties as sources of genetic diversity for breeding to support tropical production systems, improve nutrition, and boost income. One of earliest activities of the new center, beginning in 1969, was to organize the collection of cassava landraces to establish a genetic resource for breeding purposes at the center’s headquarters in the Cauca Valley. We describe the multifaceted understanding about this remarkable heritage through thorough evaluation over a diverse range of environments and the creation of new varieties aimed at adding value across the crop’s diverse agro-ecologies and uses. CIAT breeders, along with a broad coalition of partners, have targeted demands from growers, processors, and consumers throughout the tropics. Latin American germplasm remains the foundational source of novel, high-value traits that differentiate cassava products across markets, underpinning the continued gains in productivity, quality, and end-use performance.

## 1. Introduction

This paper, along with its companion Part I [1], are broad historical reviews of the cassava (*Manihot esculenta*) germplasm collection at the International Center for Tropical Agriculture (CIAT) in Cali, Colombia. Together, these reviews cover collection, conservation, characterization, evaluation, distribution, and use in variety development. This history is extracted from more than 50 years of peer-reviewed papers, institutional reports, conference proceedings, research program databases, and scientist experiences.

Progress in cassava genetic improvement owes a great debt to the early women and men farmer-breeders who developed the landrace varieties and shared them freely with germplasm collectors to become the foundation of modern breeding. Institutions like the Empresa Brasileira de Pesqusia Agropecuaria (Embrapa) in Brazil, CIAT in Colombia, and the International Institute of Tropical Agriculture (IITA) in Nigeria had the foresight more than half a century ago to organize extensive collections of these landrace varieties to save that heritage in perpetuity.

**Figure 1:**
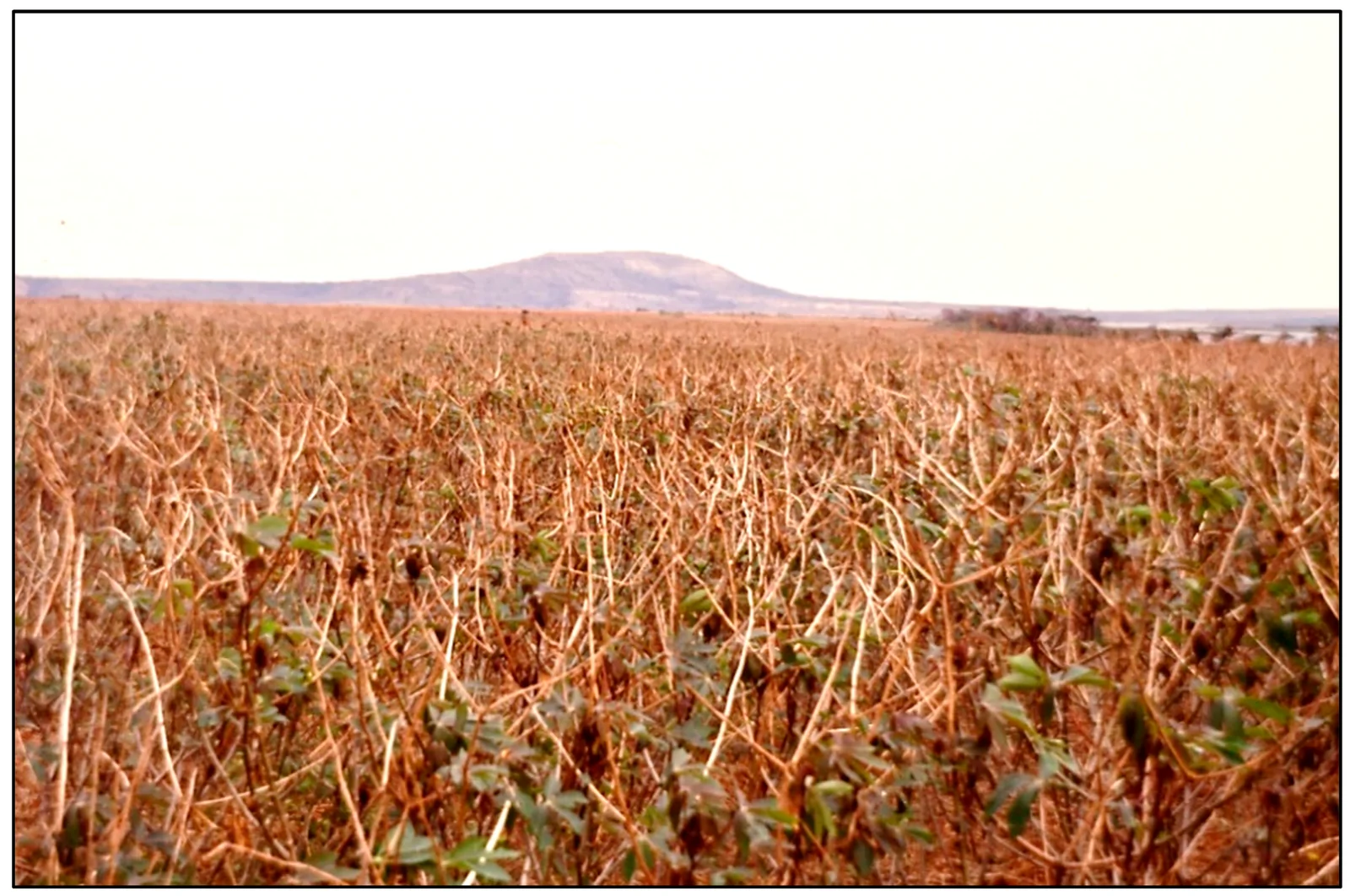
A large cassava plantation is severely defoliated by cassava bacterial blight in 1979 in Minas Gerais, Brazil. The low levels of resistance in this local variety were sufficient to control the disease in isolated low-intensity traditional production systems but not in large-scale monocultures where the pathogen spread quickly among plants. This experience typified results from moving landraces from their origins to very different environments (climate, soil, biotic constraints) and especially under intensified management conditions. Photo by C. Hershey.

Until the mid-twentieth century, the tropical crop research community commonly regarded cassava as a resilient, rustic crop that required only low inputs to achieve good yields. For example, when CIAT initiated its cassava research efforts in 1969, early program notes acknowledged that cassava had been “traditionally considered a hardy crop with few production problems” [2], a perception that had contributed to decades of underinvestment in research. However, accumulating evidence demonstrated that its apparent resilience masked a complex set of vulnerabilities. The widespread cassava mosaic disease (CMD) in Africa, occasional outbreaks of cassava bacterial blight (CBB) (*Xanthomonas axonopodis* pv. *manihotis*) (e.g., Figure 1), and the reported presence of low levels of several other pests and diseases were potential new threats. The identification of soil nutrient depletion under more intensive systems illustrated limits to the crop’s adaptation to low-fertility soils [3].

By the mid-20th century, in addition to this emerging understanding about the potential importance of pests, diseases, and crop management, cassava production began to move from subsistence to commercial enterprises in limited areas, especially Brazil and Southeast Asia, creating demand for varieties with traits that could not fully be met by local landraces. These traits included higher starch content, pest and disease resistance, greater yield potential, response to better management (land preparation, weed control, modest fertilizer use), and other traits impacting suitability for diverse markets.

CIAT understood that breeding would require stable long-term investment built on a broad germplasm base, with extensive research and development partnerships in target regions. Early visions evolved into the following framework:

- Understand the current production systems and markets.
- Define the needed changes for priority target geographies, production systems, and markets.
- Establish the short- and longer-term roles of breeding to achieve goals.
- Evaluate and understand the traits of the available germplasm base.
- Define and implement a breeding strategy jointly with appropriate partners.
- Establish and measure the metrics for success and the need for adjustment at each step of the framework.

Several reviews have summarized the broad history and accomplishments that grew out of this framework at CIAT [4,5,6,7,8,9]. The current paper tracks how CIAT’s germplasm collection, consisting mainly of farmer-developed landrace varieties from the crop’s center of origin in the American tropics, has contributed to progress in cassava genetic improvement in partnership with many national institutions worldwide.

## 2. A Framework for Discovering and Exploiting Value in Cassava Landrace Varieties

### 2.1. Historical Context

CIAT’s long history of cassava breeding passed through several major loosely defined strategic approaches, or phases. Each of these involved the evaluation and use of genebank accessions in different ways. While they are not necessarily formalized in institutional documents, the phases are distinguished by a broad-scope view presented in cassava program annual reports, available at CGSpace.org. Table 1 illustrates trends in research and development related broadly to targeted benefits, and to cassava breeding goals specifically. Certain basic priority traits like yield and starch content have been constant, but nearly all other traits have undergone various degrees of emphasis, de-emphasis, or regional re-prioritization according to changes in the biophysical, political, social, or funding environments.

### 2.2. An Evaluation and Selection Framework

Over CIAT’s 50-plus-year history, the use of genebank accessions by the cassava program has largely focused on the original purpose described by the founders: “as a source of genes to improve cassava traits of grower and consumer interest, through selection and breeding” [2]. To best achieve this goal, the management and use of the cassava collection was always closely integrated with the genetic resources and cassava programs.

The early CGIAR system especially emphasized the benefits for society from crop genetic improvement, and this early focus was applied to cassava. Phenotypic evaluation of the collection began in earnest in 1972 with the hiring of Dr. Kazuo Kawano, the first CIAT cassava breeder, a few years after the collection was established in 1969–1970 [2]. Even before the evaluation of existing landraces had begun, there was an assumption that scientific breeding could improve upon what farmers had developed as landrace varieties. Based on this assumption, and very early in the evaluation process, Dr. Kawano began making crosses among accessions that showed tentative promise—heavily prioritizing the harvest index at the six-month harvest of just three plants in a single-row trial.

From the outset, and consistently over decades, evaluations of the collection of landraces and of the hybrids generated from them were fully integrated into a unified evaluation and selection pipeline, i.e., using same testing sites and similar evaluation criteria. Only selected accessions or breeding lines continued in the evaluation pipeline, with progressively more sites and larger trials. As a result, the evaluation data across the collection of landraces were very uneven. Many accessions have only data from single-row trials in two evaluation sites. Only a relatively few continued through the successive selection stages in multiple sites for more thorough evaluation.

With various modifications over time, the pipeline broadly involved:

For hybrids only:

- F_1_ individual plants from true seeds: CIAT headquarters only, where delicate seedlings could be adequately cared for.
- F_1_C_1_ (first clonal generation; plants with minimal selection from F_1_ plants): A selection stage that was introduced to facilitate introducing the broadest possible genetic diversity to sites outside of CIAT’s headquarters.

For genebank accessions and hybrids:

- Single-row trial: An unreplicated trial, using 1 × 5 to 7 plants and 2–3 locations.
- Preliminary performance trial: An unreplicated trial, using 4 × 6 plants with border rows and 2–3 locations.
- Advanced performance trial: A total of 2–3 reps., using 5 × 6 plants with border rows and bordered plots in 2–3 locations.
- Regional trials: A total of 4 reps. used in multilocation trials, mainly on-farm.

Trial names and designs varied somewhat over time. Detailed descriptions of the breeding pipeline across the years can be found in [4,5,6,7,8,9]. In particular, Ref. [9] provides considerable detail on strategy and evaluation procedures (including ontology), which applied mainly during CIAT’s first two decades when most of the field evaluations of landraces took place. CassavaBase.org provides a more complete description of ontologies for cassava, including crop ontology entries (CO_334:xxxxxxx) and composite traits (COMP:xxxxxxx) [10]. Many of these represent canonical references for the same biological trait, differing only in measurement protocol, scoring method, or stage of crop development. Variation in trait ontologies is an expected consequence of changes in evolving breeding objectives, improved phenotyping methodologies, breeding program staff, and the progressive implementation of program-wide data governance and standardization. Table S1 provides the links between historical breeding materials and their corresponding genebank accessions, offering a practical starting point for navigating the available germplasm and associated datasets.

## 3. Early Institutional Focus on Nutrition and Safety

In the 1960s, development agencies commonly prioritized combatting human malnutrition through greater intake and higher-quality protein. The possibility of significant variation for protein content in cassava became the first goal of germplasm evaluation. Under contract with CIAT, the laboratory of nutrition at the Universidad del Valle in Cali analyzed N content of roots of the early landrace collection in 1969, as based on the commonly accepted correlation between Kjeldahl N and protein content [2,11]. From these tests, the variety *Llanera* (COL1438) from the eastern plains of Colombia (“Llanos Orientales”) showed 7.25% root protein (dry weight basis), whereas most accessions were in the range of 0.5 to 1.5%. However, this initially reported high level did not hold up. The estimate of protein based on the conventional method of 6.25 × total nitrogen did not seem to be reliable for cassava, which was in fact also confirmed in later studies [12,13]. Furthermore, for starch extraction, cassava’s low protein content is generally considered a positive attribute. Consequently, this early focus on higher protein was soon discontinued.

There were also early concerns about the toxicity of “bitter” varieties, long known by cassava growers and consumers and by the scientific community by the mid-1800s. Cassava produces linamarin, a glucoside, that releases HCN when tissue damage brings the enzyme linamarase into contact with the glucoside. CIAT’s 1970 annual report noted: “sweet or non-toxic varieties have a content of less than 50 mg of HCN per kilogram of fresh roots, while bitter, toxic types have well over 100 mg per kilogram of fresh root” [11]. As with root protein levels, the focus on toxicity was short-lived. Both indigenous and more modern processing procedures effectively managed toxicity (cyanogenic potential (CNP)). CIAT shifted away from the idea of a broad-based lowering of CNP, but it did continue the selective breeding of low-CNP types targeting consumption directly after boiling. For highly processed roots either for food or industrial use, or even for starch extraction, the CNP of raw roots was much less of a concern.

## 4. The Search for Yield Potential, High Starch Content, and Broad Adaptation

**Figure 2:**
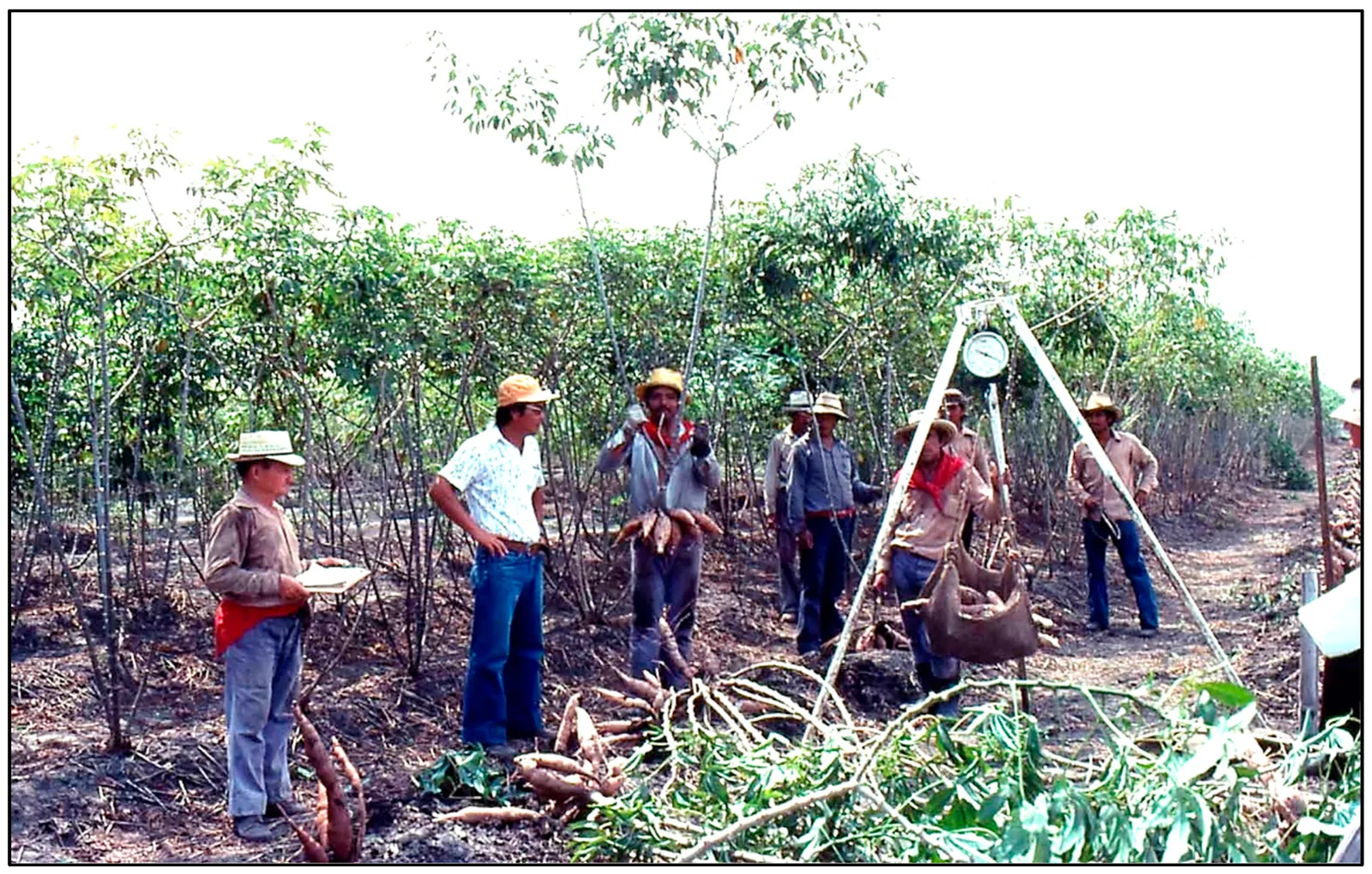
Kazuo Kawano (second from left), CIAT’s first cassava breeder, leads his team in early evaluation of the genebank accessions for agronomic traits in 1974. (Photo by CIAT).

With the arrival of CIAT’s first cassava breeder in 1972, the evaluation of the newly established collection shifted toward understanding the diversity of key agronomic traits (Figure 2). This focus was strongly influenced by the Green Revolution’s successes in wheat and rice in the 1960s, where breeders improved yield potential mainly by increasing the harvest index—the proportion of grain to the whole-plant weight for grains, or harvested roots relative to the whole-plant weight (roots plus leaves and stems) for cassava.

Additionally, the starch content (closely related to dry matter content (DM)) was a top priority, as it is cassava’s principal nutritional or industrial product. Starch normally comprises about 85% of cassava’s dry matter content.

One of the breeding team’s first activities was to plant the entire collection of about 2000 accessions at the CIAT-Palmira headquarters station in 1973, in single-row unreplicated trials. While several morphological traits were evaluated, especially those related to plant architecture (height, branching habit), nearly all the focus for selecting an initial set of 230 promising accessions was based on the root dry matter content, yield, and harvest index [7] (Table S2).

In the following season in 1974, this group of selected accessions was planted in a replicated yield trial in the headquarters station, and in single-row trials at two stations believed to be more representative of farmers’ conditions. The ICA-Carimagua research station of Eastern Colombia (Meta Dept.) is characterized by highly aluminum-saturated soils, a rainy season that favors severe disease development, and a long dry season. The ICA-Caribia station in the Atlantico Dept. of Northern Colombia has a seasonally dry, hot climate that especially challenges the expression of high, stable starch content, and favors high cassava green mite (*Mononychellus tanajoa*) populations.

However, by this early phase of evaluation, the main interest had already shifted to developing hybrids using the early headquarters selections rather than a more comprehensive and precise evaluation of the landraces. The group of 230 selected accessions (mostly landraces) formed the early core breeding stock for the program. In a review of the first 30 years of cassava breeding at CIAT, Dr. Kawano noted: “The great initial germplasm variation seen in the 1973 CIAT headquarters single row trials set the basis for the overall progress during the following 30 years” [7]. This assessment was later amended in recognition of the diversity for adaptation, resistance, yield, and quality traits that had not yet been evaluated across contrasting environments, leading to the third phase of germplasm evaluation over a broader range of environments (Section 5).

Table 2 lists the group of landraces that the breeding section identified over the following few years as the most promising parents for developing new hybrids among these 230 accessions. Just three countries contributed parental materials: Colombia, Mexico, and Venezuela. During this time, Brazil, commonly believed to be home to the most diverse array of landraces, was represented in the genebank by only a few accessions due to concerns about introducing coffee rust spores along with cassava stem pieces [1].

In these first years, CIAT saw the continuing evaluation of landraces as a less promising pathway for identifying superior parents, as compared to a recurrent selection scheme based on the progeny from the initially selected group. In 1974, the program produced about 35,000 seeds from controlled crosses using 250 different cross combinations [14]. Although pollination is labor-intensive, the breeding program produced considerably more hybrid seeds than it could manage in local trials. Others were shared with the growing cadre of partners, mainly in Latin America and Asia. About 15,000 open-pollinated seeds, collected from 203 promising accessions, were also sent to IITA in Nigeria.

While much of the earliest hybrid seed production from landraces was aimed at establishing methodologies and training partner breeders, the genetic foundation was also critical to future progress. This was especially true of the early developing partnerships in Thailand, the Philippines, and Malaysia. Asia in the 1970s (except for India) had a very low level of pest and disease pressure on cassava. Most introduced materials survived, and the selection for yield potential and high dry matter progressed relatively quickly [7].

Thailand was a unique example of a situation in which breeding advanced rapidly. First, much of the country’s traditional production was based on a single local variety, *Hanatee* (TAI9 in CIAT’s collection). This variety is a likely duplicate of various other landrace accessions from different regions that dominated fresh consumption markets, combining moderate yield and starch content with favorable cooking time and strong consumer preference. Secondly, in the 1960s and 1970s, Thailand rapidly expanded cassava production driven by policy distortions that increased the demand for dried cassava chips and pellets in animal feed markets in Europe. Production remained almost entirely with small farms of a few hectares or less. The stringent, complex quality requirements for human consumption (especially of boiled roots) were not part of the equation. Breeders could focus almost exclusively on yield and starch content. Over time, other traits entered the product portfolio, but for many years breeding in Southeast Asia was less complex than for many other production environments and market conditions.

It is certainly plausible, and in fact likely, that in the first two decades of existence of the cassava germplasm collection, CIAT introduced a substantially greater range of genetic diversity—mainly through seed progeny from genebank accessions—to both Africa and Asia than had been distributed to these continents in the previous 500 years. Instead of the introduction from limited geographies in colonial times, CIAT’s new collection spanned large parts of cassava’s production range throughout the Americas.

## 5. Revisiting the Landraces to Target Diverse Edapho-Climatic Zones

### 5.1. Strategy Development

There had been excellent progress in demonstrating genetic gains for the yield potential and harvest indices from the early crosses, as reported in CIAT’s cassava program annual reports (accessible on CGSpace.org). But the program recognized that the germplasm base from which this progress was derived, while diverse, was likely to be somewhat narrow relative to the total diversity of the species. For example, only three source countries were represented, and with pre-selection at the highly productive CIAT headquarters station.

While broad adaptation was an early breeding goal, by the mid-1970s, there was growing evidence of strong G × E interactions for many landraces when planted across a range of cassava-growing environments [15]. Landraces often responded very differently in environments that contrasted in climate, soils, and/or pest and disease complexes. This led to a modification of the early evaluation scheme to begin prioritizing diverse off-station sites within Colombia. These trials were critical in revealing some traits that were not possible to evaluate at CIAT headquarters, such as acid soil tolerance and resistance to CBB or superelongation disease (SED: *Sphaceloma manihoticola*) in the eastern plains; adaptation in a subhumid, hot environment in the Colombian northern coastal region where cassava green mite pressure was often high; or in the high-altitude, cooler climates of the Andes.

Breeders found that it was not practically feasible to combine resistance or adaptation to all the important constraints or conditions across the crop’s many production environments while at the same time selecting for high yield, good architecture, good root form, high starch content, and an array of other preferred traits (see examples in Table 3). Some subdivision of breeding objectives was required to make significant genetic gains. However, we also know from recent genotyping data that some landraces have historically spread widely across countries and even continents [16]. These varieties have shown broad geographical range, but they were not necessarily adapted to a broad range of biotic and abiotic influences on growth and development.

These evidence-based conclusions led to a notable shift in strategy. In 1978, the program initiated a focus on “germplasm development”, i.e., to further explore the potential to exploit a broader range of diversity of landraces. The genebank was continually being augmented since the first collections back in 1969–1970. Notably, by this time, over 1000 accessions had been introduced from Brazil and other countries and regions with expected important diversity, such as Peru, Ecuador, Paraguay, Central America, and the Caribbean [1].

At this early stage of cassava’s genetic improvement, there was typically not a large gap between the appearance or performance of landraces as compared to breeding lines or released varieties. As a result, crossing best hybrids with landraces could still result directly in progeny with superior performance. This contrasts with many modern crop varieties, where “pre-breeding” is required to gradually introgress target traits from landraces into progressively more agronomically adapted types over a few or several breeding cycles.

Under this new germplasm development initiative, beginning in the late-1970s, CIAT’s breeding program (which also curated the field genebank) began an extensive new evaluation of the full collection of landraces across diverse sites outside CIAT’s headquarters. In general, the off-station sites were selected to represent key agro-ecological characteristics of broadly defined major production areas of the world or areas with high potential for expanded production. CIAT selected and managed these evaluation environments to provide target physical and biological stresses at moderate-to-high levels in order to have the best opportunities to observe target trait expression. Under this scheme, trials were managed with good but not luxurious agronomic practices. Pests and diseases were rarely artificially controlled in order to identify and improve genetic resistance or to optimize conditions for biological controls.

**Figure 3:**
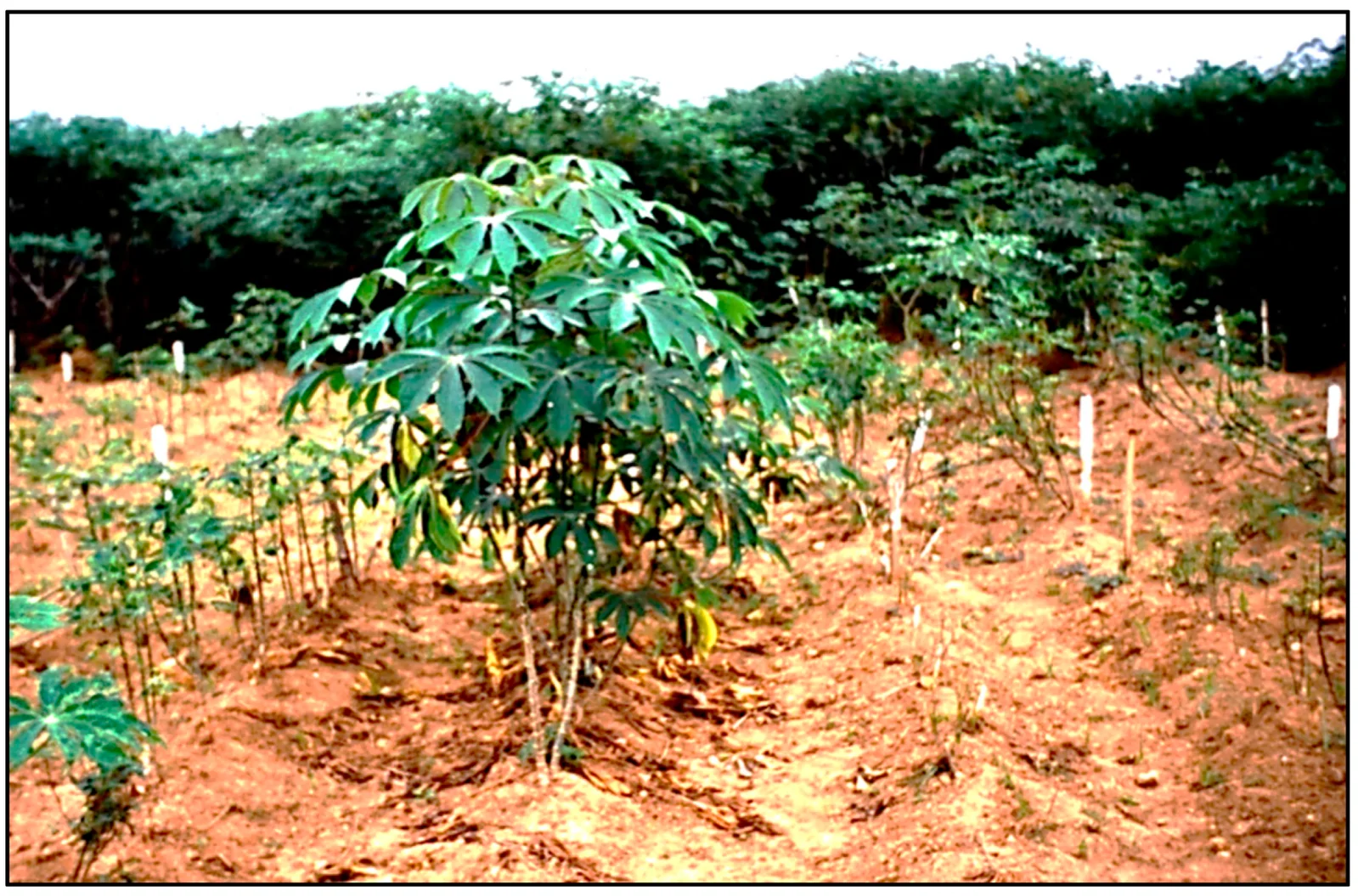
In the semi-arid region of Colombia’s Guajira Peninsula, cassava green mite (*Mononychellus tanajoa*) populations can be very high. Many genebank accessions barely survive these combined drought and pest stresses. In comparison, an adapted resistant landrace from the germplasm collection is shown in the foreground.

In a typical growing season at the headquarters station, nearly all genebank accessions were able to prosper reasonably well, i.e., to result in plants with the ability to clonally reproduce well through good-quality stem cuttings while producing at least a modest root yield. On the other hand, at the off-station sites, many accessions did not survive the entire growing season or were very weak with little to no yield (Figure 3, Table 3). This differential performance was key to identifying adaptation and resistance traits in the collection that would subsequently prove invaluable for breeding.

Based on this decade of experience (late-1970s to late-1980s), the program defined combinations of biotic and abiotic conditions broadly representative of global cassava production [15]. These came to be known as “edapho-climatic zones” (ECZs), as they were defined fundamentally by a combination of soil and climate criteria believed to be of most consequence for cassava’s adaptation and success [9,15,17]. The description of ECZs was specifically oriented to cassava’s adaptation and constraints (biotic and abiotic). Therefore, it did not coincide directly with any standardized soil or climate classification. Nevertheless, Carter [18,19] proposed approximate equivalencies between the standardized classifications of climate and soil restrictions and the cassava program’s ECZ descriptions. CIAT illustrated an approximate mapping of ECZs in the Americas, overlaid with the density of cassava production [20].

Most of the landraces were evaluated in field trials in four edapho-climatic zones in Colombia (ECZs 1, 2, 4 and 5), whose main features are listed in Table 4. More limited numbers were evaluated in ECZs 3 and 7, and none were evaluated in ECZ 6 due to the fact that no parts of Colombia extended to the subtropics. Generally, initial evaluations consisted of just one year within each site, where plots were single, non-replicated rows of 5–7 plants.

With the initiation of a strategy to develop populations (also described as gene pools) for broadly defined ECZs, there has been strong attention given to targeting pest and disease resistance and soil and climate adaptation. The goal shifted from measuring yield potential under favorable conditions of experiment stations to integrated performance under conditions that farmers more typically encounter.

The program aimed at balancing resistance, soil and climate adaptation, quality, and productivity within distinct (but loosely defined) gene pools. Breeders recognized this to be a long-range goal, one built on landrace varieties that individually contributed in partially and complementary ways to the overall complex of ECZ-targeted traits.

The relative equivalency of these ECZs among countries was difficult to test in the field. Establishing international uniform performance trials with statistically rigorous analysis would have been invaluable for fine-tuning the ECZ definitions across countries. But for cassava, a long-season clonal crop with many quarantine constraints, this is slow, complex, and costly, and was not prioritized by donors. Instead, the general relevance of CIAT’s ECZ classification was supported by the evaluation of non-uniform diversity across cassava-growing environments outside of Colombia. Typically, however, national breeding programs are oriented to a much narrower range of edapho-climatic conditions than CIAT is as a global provider of diversity. The need for this broad range of target descriptions is essentially unique to an international center like CIAT or for large countries like Brazil.

### 5.2. Pest and Disease Resistance

Sites selected for evaluation were chosen to have a balanced mix of pests and diseases representative of major global production areas. However, these biotic pressures will always vary from one year to the next and among micro-ecologies within a region. Population levels and their uniformity could be partially managed by techniques, such as the choice of planting date or planting susceptible spreader rows. In an effort to improve the effectiveness of selecting for resistance, CIAT developed parallel methodologies for greenhouse and screenhouse evaluations, using young potted plants or detached leaves. There were some successes, but the correlation between greenhouse and field evaluations was often marginal. The general conclusion was that any tentative resistance found under artificial growing conditions needed to be confirmed in field trials as early as possible in the breeding pipeline. There could be interactions with many growth and development factors that impacted the expression of resistance [17].

Parsa et al. [21] provided a useful model for interpreting and using landrace evaluation data through a meta-analysis of trials established between 1980 and 2004. They included 31 single-row trials, 27 preliminary yield trials, and 31 advanced yield trials. The study’s goal was to identify promising sources of host plant resistance for use in breeding. The data included the evaluation of damage levels from cassava green mites, cassava whiteflies (*Aleurotrachelus socialis*), and thrips. The results showed that natural populations, with appropriate experimental design, can create an effective system for identifying resistance. With appropriate planning between breeders and plant protection specialists, resistance evaluations under natural infection/infestation conditions can be part of a comprehensive system for evaluating many traits. The choice of appropriate selection sites is also fundamental.

Parsa et al. [21] also considered but discarded several other pests in the resistance study. Although some 3000 accessions had been evaluated for mealybug (*Phenacoccus herrenii*) resistance, there was little variation in the damage levels among clones [22,23]. Other studies have sampled only a small part of the germplasm collection, mainly for methodology development and exploratory analyses, e.g., for burrowing bugs (*Cyrtomenus bergi*), fruit flies (*Anastrepha pickeli*), gall midges (various Cedidomyiidae), grasshoppers (various Orthoptera), lacebugs (*Vatiga illudens* and *V. manihotae*), scales (various Coccoidea), and stem borers (*Chilomima* and *Coelsternus* spp.).

An important lesson from the germplasm evaluations is that a preliminary evaluation for pests and diseases (e.g., single sites with no replication) should be considered only as a rating of location/year-specific “damage levels”. They should not be called or considered to be “resistance” ratings until further confirmed by trials appropriately designed for statistical analysis. In this sense, most pest and disease data available from genebank accession evaluations included in the Genesys-pgr.org database should be considered as preliminary and interpreted as damage ratings rather than resistance ratings.

### 5.3. Quality Traits

The evaluation of landraces by the breeding team typically included measures of very basic quality traits, such as starch/dry matter (the specific gravity method) and cyanogenic potential (the picrate method). Because cooking trials are time- and labor-intensive, they were normally conducted only at CIAT’s headquarters and only for accessions that had progressed from the selection stages through to more advanced trials. By the late-1980s, CIAT’s market studies concluded that the creation of dual-purpose varieties (industrial and human consumption, especially for the fresh market) was not likely to be an efficient breeding strategy. Maintaining the many traits that consumers prefer while simultaneously improving yield and resistance is highly complex. A breeding goal of dual-purpose varieties would create an untenable drag on the much easier goal of improving the yield potential in varieties where starch content alone is the main market consideration. Following these early market studies, the breeding program divided each of the ECZ-oriented gene pools into a low-cyanogenic-potential (CNP) subset (fresh-market-oriented) and a subset for which CNP was not considered during selection (highly processed for food, or for industrial uses) [9].

However, until recently, most of the breeding effort has been led by the industrial (Asia) and processed food markets (Africa), with lesser attention to fresh-market quality. As a result, the database for fresh-market quality is somewhat limited for the germplasm collection. From 1999 to 2003, the Cassava Quality Laboratory conducted extensive trials across a range of traits (Table S3). In these trials, “ease of cooking” includes cooking time and texture (mainly softness or hardness), but this does not include other factors that may be important to direct consumption, such as taste or “mealiness”.

In recent years, there has been good success with near-infrared spectroscopy (NIRS) in estimating several starch and structural components for cassava (along with other root and tuber crops). The RTBfoods project, managed by CIRAD (France) and focused on food uses in Africa, identified and prioritized quality traits for different products and markets, as well as the potential high-throughput tools for their evaluation. Two special issues of journals included several papers on high-throughput screening techniques to measure and improve consumer quality for cassava [24,25]. These priorities and tools have already been applied on a limited basis to CIAT’s cassava landrace collection and should provide new approaches that warrant reevaluation [26].

### 5.4. Superior Accessions Incorporated into the Breeding Pipeline

Based on the ECZ adaptation strategy from the late-1970s onward, all accessions of the genebank were evaluated across a diverse range of sites rather than preselected at CIAT’s headquarters, as was the case in the first years of the breeding program. This change identified considerable new diversity of value, especially for resistance to multiple pests and diseases, adaptation to seasonally dry conditions, adaptation to acid soil conditions, and tolerance to the low temperatures of the Andean highlands. Table 5 summarizes evaluations by ECZs from 1980 to 2023.

Compared to the few source countries of key parents from early crosses (1970s), this expanded search for traits across ECZs drew much more broadly across the germplasm collection (Table 6). In particular, accessions from key diversity centers in Brazil were now included more extensively.

During this second phase of breeding, the genebank contributed about 255 accessions to new cross combinations, or just over 5% of the total collection. While these numbers indicate that a large part of the collection remained untapped, they reflect extensive use of a broad genetic base relative to what many crops achieve in mainstream breeding programs. This more extensive revisiting of the germplasm collection to target multiple constraints of diverse ECZs continued for several years into the mid-1990s.

Each of the broad-based gene pools was managed as a loosely defined breeding population. While most crossing occurred within each gene pool adapted to a given ECZ, there was also considerable interchange among them, especially where there were shared constraints across ECZs. Beyond the 1990s, there was limited return to the genebank to screen for broad-based adaptation to the individual ECZs. Focus turned to improving specific agronomic, resistance and quality traits.

As breeding progressed over the next few decades, an elite group of accessions became distinguished by the frequency of their use in the development of superior hybrids, often through complex pedigrees (Table S4). For example, twelve accessions were used in over 100 different cross combinations to supply CIAT and its partners with high-value progeny for variety development (Table 7).

During this era of breeding, a select group of landrace accessions and hybrids were designated with an “elite” status. Elite clones were the most promising based on multi-year, multi-location trials in diverse sites across Colombia fitting descriptions of ECZs 1 to 5. Few trials were conducted in the tropical rainforest environment, due in part to the lower demand for new varieties in traditional cultivation systems at that time and because of security challenges in the area (Table 5). Most were indicated for their performance in a specific ECZ, but many also performed well across two or more ECZs. These elite clones formed the basis for most of the hybrid seeds sent to partners over the next few decades. Table S5 summarizes the wide array of landraces that participated in developing these ECZ-targeted gene pools. Some notably strong performing accessions emerged from this process (Table 6).

Table S5 shows a complete list of genebank accessions that became progenitors of these elite hybrids. Many clones were able to contribute across the ECZs and outside of their principal zone of best overall performance. A small set of clones had genebank components from across four or five ECZs (Table S5). This illustrates the breeding strategy of focusing initially on a set of core traits needed for specific ECZ adaptation, while also bringing in new traits from a diverse range of accessions to strengthen the overall gene pool. One of the notable features of Table S5 is the very low frequency of accessions of Brazilian origin that participated in the genealogies. BRA 5, BRA12, and BRA35 were key exceptions. The reasons are unclear without further study. It may be that the delayed introduction of Brazilian landraces (such as the quarantine issues described earlier) delayed their evaluation and eventual potential use as parents; alternatively, the continuing evaluation and analysis of the genebank may have possibly shown that greater diversity evolved outside the presumptive evolutionary homeland of the crop [1]. In the highlands group (ECZ 5), nearly all accessions except for two were of Colombian origin, showing the narrow range of low-temperature tolerance.

Table S6 shows more broadly a comprehensive list of over 40,000 crosses made over CIAT’s history based almost entirely on a genealogy of genebank accessions, including links to additional clone information in Cassavabase.org.

Several factors came into play during the implementation of the ECZ-oriented strategy to bring greater diversity from the landraces into the mainstream breeding pipeline. By the mid-to-late-1990s, there was good success in exploiting new traits from the genebank. The strategy had resulted in many elite clones with good ECZ-oriented climate and soil adaptation, yield, and reistance traits, which became the basis for ongoing breeding progress (Table S4). ECZ 2 (acid soil savannas, represented by the Carimagua station in the eastern plains of Colombia) is a good example of this. In the initial evaluation of the landraces in Carimagua in the mid-70s to early-80s, many accessions were killed off by bacterial blight or superelongation disease in the rainy season, and further stressed by green mites, lacebug, or stem borers in the dry season. Only a limited few produced commercial yields (e.g., in the range of 10–15 tons per hectare equivalent). After a few generations of selection and recombination, new hybrids routinely performed well in this environment, e.g., according to CIAT’s annual reports between the 1980s and 1990s (available on CGSpace.org). The same experience applies to the tropical highlands ECZ. Initial landrace evaluations showed a very low frequency of adapation, but progress after a few cycles of recurrent selection generated many adapted hybrids; meanwhile, less extreme outcomes were observed in ECZ 1 (subhumid lowland tropics). As the most common agro-ecology for cassava, many landraces were reasonably well-adapted.

The continuing introduction of new accessions into the genebank had considerably slowed by 1990 [1]. Under budget constraints broadly across CGIAR, the cassava program began to re-focus on just the highest priority targets, especially in Southeast Asia. Brazil, with almost three-quarters of Latin American cassava production, had both a very broad native genetic diversity and strongly supported genetic resources and breeding programs. Many other programs that had once been strong CIAT partners were seeing diminished government support—Mexico, the Dominican Republic, Panama, Ecuador, and Peru, for example—and institutional demand for new germplasm in Latin America fell sharply. At the same time, new interest in the Caribbean created new demand, and CIAT, mainly through the Latin American Cassava Consortium (CLAYUCA), shared many accessions and hybrids for evaluation [27].

With these ECZ-oriented hybrids as components of core gene pools, including traits that had been enhanced and combined from their landrace backgrounds, it was now possible to continue investigating genetic advances with a stronger focus on country- and region-specific traits requested by major partners. In fact, we do not know whether the initial narrowing of the genetic base by the selection of just 230 accessions under CIAT’s headquarters’ favorable conditions created any significant bottleneck to future breeding progress. Further analysis and comparison at the genomic level should provide insights. It does appear that, in spite of this initial limited selection of landraces at CIAT headquarters, there was enough diversity—and even resistance to several untested pests and diseases—to allow for significant contributions to partner breeding programs globally.

## 6. Frameworks for the Strategic Use of Diversity Under Multilateral Coordination and the Integration of Modern Genomics

### 6.1. Evolution of Partnerhips in Asia and Africa

By the 1990s, there was relatively little new direct use of genebank materials in breeding. In Asia, partner breeding programs in major cassava-growing countries had been considerably strengthened since their early development. They were well-equipped to manage segregating populations from crosses of promising parents. The main interest had shifted quickly from receiving “finished” varieties as clonal material to receiving seed progeny from parents whose traits complemented the diversity that was available locally. High and stable starch content, high yield potential, and erect plant architecture often topped the list. The genebank had already been broadly tapped for these traits, and most breeders focused on continuing to advance and fine-tune populations rather than introduce more landraces.

With few exceptions, CIAT’s distribution of germplasm to Africa was through IITA. Inter-center collaboration between IITA and CIAT remained informal and unstructured for many years. The cassava programs interacted mainly at the level of individual scientists, especially with strong collaboration in entomology and breeding.

Quarantine restrictions on moving clonal materials, in both directions between Africa and the Americas, meant that there was very limited exchange of landraces. In the late-1980s, CIAT posted a liaison scientist, Dr. Marcio Porto, at IITA, and a key part of his responsibility was to support the integration of additional Latin American germplasm into Africa. During this period, CIAT introduced approximately 130,000 seeds from 725 families for testing by the IITA breeding program, with an aim of expanding the genetic base for new variety development [28]. Nearly all these seeds had Latin American landraces from the genebank in their pedigrees. Many seeds were the progeny from crossing Latin American landraces onto cassava mosaic disease (CMD)-resistant lines introduced to CIAT from IITA in the mid-1980s [1].

As expected, most introductions, especially those that did not include sources of CMD resistance, would fall to CMD. Evaluations also focused on other traits of interest relevant to Africa, such as high starch content, high yield, erect plant type, low cyanogenic potential, and resistance to cassava green mites [28]. According to reports from Dr. Porto’s team, CIAT’s introductions did produce some promising candidates for further breeding—importantly, including some that combined resistance to CMD, cassava bacterial blight, and cassava green mites. The studies also confirmed that selections from the edapho-climatic zones defined in Colombia were relevant for the areas with similar climate and soil conditions in Nigeria [28].

However, the follow-up records of further performance or use of these materials are not readily available. Although the specifics are unknown, these crosses are likely to have contributed to successful introduction of new diversity in Africa. With additional data from the landrace and hybrid genotyping at both IITA and CIAT, more of this story should be revealed. In 2025, CIAT and IITA formalized some of their key inter-center collaborative interests through a memorandum of understanding [29].

### 6.2. Project-Oriented Funding and Platforms for Inter-Institutional Communication and Coordination

Beginning in the late-1980s and 1990s, CGIAR funding began to shift from a core pool committed to continuity and stability to higher dependence on special projects that tended to have a 3–5-year life span. This framework challenged the steady long-term funding required for the management of genetic resources and breeding. CGIAR, as a system, as well as individual centers, responded by undertaking a series of initiatives to enhance research effectiveness. Many were aimed at boosting inter-institutional collaboration and targeting intransigent research bottlenecks, especially through the application of modern genomics. None of these multilateral projects or initiatives were specifically focused on the evaluation and use of landrace varieties from the Americas, but some did support those activities as part of broader objectives. Some key examples are summarized here.

CIAT established the Cassava Biotechnology Network (CBN), which had its core period of activity from 1988 to 2004. CBN supported many genetic resources and breeding-oriented projects, as well as six international scientific meetings [30]. The proceedings are available via CGSpace.org.

In 2002, CIAT led the launch of the Global Cassava Partnership for the 21st Century (GCP21). Its purpose was to coordinate and accelerate global cassava research—especially modern biotechnology and breeding—to make cassava more productive, resilient, and beneficial for food security and economic opportunity. In its most active years, it organized regular international conferences and supported many projects that involved genetic resources [31]. The CBN and GCP21 marked a decisive shift toward multilateral and globally networked cassava research.

The CGIAR Generation Challenge Program (GCP), initiated in 2003, expanded work on genomics-enabled breeding, especially emphasizing molecular markers, trait mapping, and the integration of landraces into modern breeding pipelines. The GCP renewed interest in landraces, especially in Africa and the Americas, and brought together a broad range of expertise and resources to better collaborate among centers and national program partners [32,33].

The HarvestPlus initiative, which began in 2003, supported breeding to improve crop key nutrient contents. For cassava, the main focus was to deliver better nutrition for vitamin-A-deficient populations in Africa [34]. CIAT’s main participation was the introduction of new sources of enhanced beta-carotene content, including sources from landraces [35].

In 2012, CGIAR organized the CGIAR Research Program on Roots, Tubers and Bananas (RTB-CRP) to exploit the capacities and efficiencies of multi-institutional collaboration on shared challenges and opportunities among root and tuber crops. One of its stronger initiatives was based on a recognition of the need to pay greater attention to user preferences for root quality for human consumption, including traits that may have differential preferences between men and women. The root quality screening of the germplasm collection, which had begun many years earlier (Genesys.org and Table S3) [26,27,35], was a valuable resource. CGIAR and national breeding programs focused on specific product profiles based on their target geographies and end users, as well as their comparative advantages in breeding.

NextGen Cassava, led by Cornell University, modernized breeding through genomic selection and the Cassavabase.org data systems, focused on Africa. A key role of CIAT in this project was to explore avenues for enhanced flowering in cassava. The discovery of improved flowering through LED simulation of extended photoperiods opened pathways to facilitate greater integration of poorly flowering landraces into breeding populations [36,37].

A recent iteration of CGIAR’s system-wide breeding-focused platforms is Excellence in Breeding (EiB), including the data platform Enterprise Breeding System [38,39].

Taken together, these developments mark a decisive evolution in CGIAR’s impact on cassava improvement strategy. The elements of the global cassava improvement system have evolved toward a highly networked model grounded in multilateral projects. This research massively expanded the attention to cassava as a crop of scientific interest. Hundreds of published papers and institutional reports have come from these initiatives and their spin-offs, and are easily accessed via web searches and AI tools.

### 6.3. Overview of the Application of Genomics Tools

Early applications of molecular markers have focused on developing genomic resources, including microsatellite (SSR) markers for germplasm characterization, genetic diversity analysis, linkage mapping, and pedigree verification [40,41]. These resources have subsequently supported trait mapping for carotenoid content, cassava mosaic disease resistance, and starch quality. Marker-assisted introgression has been applied to a limited number of major-effect loci, including CMD2 for cassava mosaic disease resistance [42,43,44].

The integration of molecular markers and genomic technologies into CIAT’s cassava breeding program has occurred relatively recently within the context of more than five decades of variety development. For most of this period, genetic improvement relied on recurrent phenotypic selection, multi-environment evaluation, and the use of the germplasm collection as a source of new phenotypic variation. Emphasis has shifted from marker discovery to marker deployment through the validation of marker–trait associations across unrelated populations and the development of breeder-friendly competitive allele-specific PCR (KASP) assays for routine breeding applications. One example is the development and validation of KASP markers for whitefly resistance for use in marker-assisted selection [45]. In parallel, CIAT and IITA’s co-development of a mid-density SNP marker panel for cassava (available through the Excellence in Breeding platform: https://excellenceinbreeding.org/toolbox/services/mid-density-genotyping-service (accessed on 12 July 2026) has facilitated the broader integration of molecular tools into breeding workflows. The application supports parentage verification, genomic relationship estimation, quality control, genomic prediction, and genomic selection. Within the historical timeframe of this review, these approaches have contributed to a relatively small proportion of the use of landraces, reflecting a comparatively recent implementation within routine breeding operations.

## 7. Landraces in Product Profile Pipelines

Since 2021, CIAT has operated dedicated breeding pipelines for: fresh-market cassava, targeting improved cooking quality and low CNP; processing/industrial cassava, targeting high and stable starch yield and erect plant type; biofortified cassava, targeting increased carotenoid content for fresh and processed markets; and specialty starch cassava, targeting low amylose content aligned with industrial starch requirements (Table 7).

The program is now undergoing a transition from phenotypic recurrent selection to an inbred-hybrid strategy [46]. An initial focus targeted the recovery of CMD and CBSD resistance in improved backgrounds aligned with both fresh-market and industrial sectors. Over this transition period, ~25% of all crosses have involved genebank accessions, highlighting their role as a key source of market-differentiating traits (Table 7). In parallel, 48% of these genebank accessions have corresponding self-derived (S_1_) populations, supporting targeted trait discovery and deployment (TD&D) activities across multiple priority traits (Table S7).

This strategy reflects a shift toward a long-term breeding framework that simultaneously addresses emerging pests and diseases while delivering products aligned with current target product profiles across global market sectors. Efforts have focused primarily on CMD and CBSD resistance, with ~46% of the total botanical seeds derived from genebank accessions allocated to CBSD resistance recovery in improved backgrounds for fresh and processed markets. Complementary efforts have targeted tolerance to post-harvest physiological deterioration; cassava frogskin disease resistance; abiotic stress adaptation; and resistance to key pests, particularly whitefly, thrips, and red mite (Table S7).

While these activities have largely relied on forward breeding, the program is shifting to an integrated approach that combines introgression and discovery, to enable systematic backcrossing and improved management of conversion populations through partial inbreeding in the future. This approach strengthens CIAT’s role as a pre-breeding and TD&D platform, both by sharing botanical seed populations and by fixing key resistance alleles in self-derived families, thereby improving their use in parent selections. It also supports product extraction by enabling more precise assembly of complementary haplotype mosaics in a test–cross hybrid design.

In addition to its core activities in Cali, Colombia, CIAT’s breeding program operates jointly with partners in Vietnam. Crossing efforts focus on developing competitive industrial starch clones with CMD resistance and on preemptive breeding for CBSD. Contributions from IITA’s elite CMD donors have supported this work, with evidence of favorable heterotic patterns when crossed with Southeast Asian germplasm (S. Fenstemaker, unpublished data). While much of this germplasm derives from regional elite breeding efforts, the emerging pest and disease pressures continue to necessitate the targeted use of landrace germplasm, particularly for preemptive CBSD breeding.

Parallel efforts are underway to develop CMD-resistant materials for both fresh and processed markets in Indonesia and the Philippines, where cassava plays an important role in the snack food industry. These activities have renewed interest in Southeast Asian landraces that are well-represented in local value chains and can serve as a relevant germplasm base. DNA fingerprinting analysis shows that many popular landraces throughout Southeast Asia used for fresh consumption are potential genetic duplicates to landraces from South America [1,16]. The possibility that one or more of these varieties may also be widespread in Africa is currently being verified. This not only demonstrates the relative consistency in product profiles within relatively similar agro-ecological homologues across the continents but also the potential for a global cassava strategy, where priority breeding objectives for one region can be important when preemptively breeding for future requirements for resistance traits. This relies on easy and safe germplasm and data exchange between breeding programs.

Regional breeding networks, harmonized testing protocols, and the maintenance of core germplasm collections underpin preemptive breeding for emerging pests and diseases. The coordinated strategy between CIAT and ITTA provides a framework for annual crop risk assessment, structured germplasm information sharing and decision-making, shared genotyping services, and alignment of global breeding strategies for breakthrough products.

## 8. Deep Dive into the Genebank: Examples of the Discovery and Use of Landraces to Address Arising Challenges and Opportunities

### 8.1. Background

The collection of cassava landraces at CIAT has steadily fulfilled its potential to contribute genetic building blocks for new varieties. Strategies for exploiting these resources have continued to evolve over 50 years. Breeders have achieved improvements in an array of traits to meet grower and consumer needs through mainstream breeding pipelines, all within a framework of programmed broad improvement of agroclimatic adaptation, architecture, yield, resistance, and quality. But breeders have been periodically faced with other specific challenges or opportunities that required more targeted approaches.

Pests and diseases had to be tackled on an emergency basis due to new introductions into a region or epidemics caused by a combination of factors. Opportunities driven by industrial demand for new starch functional properties led to targeted research and specific tools to unlock genetic solutions. The direct consumption market presented an array of challenges that needed to be better understood or required new tools for discovery.

Some desired traits were not found, or had not been improved to sufficient levels of expression in advanced breeding lines. A re-exploration of the landraces often played a role. Sometimes these traits had previously gone unnoticed by breeders because they were rare or hidden (e.g., traits expressed only in a homozygous recessive condition) or were especially difficult to measure.

The future will bring new challenges and opportunities not yet foreseen, or at least for which genetic diversity is still unknown. Breeding cassava involves significant lags—from trait discovery to the development of an acceptable variety that meets farmer, processor, and consumer requirements, as well as to scaled deployment needed to achieve benefits. Maintaining parallel global foresight and market intelligence to anticipate and invest in future threats and opportunities is critical to ongoing breeding efforts to maintain productivity well into the future.

This section describes a range of examples in which breeders have explored and successfully exploited landraces to address these specific challenges and opportunities. Table 8 describes the categories of traits discussed in this section.

### 8.2. Examples of Traits from Landraces and Their Utilization

Table 9 lists specific examples of traits addressing challenges and opportunities that have arisen, where some progress has already been made. They are described further below.

Cassava is the second-most important source of starch worldwide after maize. However, in contrast to the wide variety of useful starch mutants identified in maize [59], none had been reported in cassava prior to 2006 [60,61]. For the starch industry, products with altered functional properties became highly desirable [62]. Similarly, the bioethanol industry would benefit from starches that are more readily hydrolyzed, thereby reducing energy and/or enzyme requirements during processing [62]. The use of dried chips for animal feed would benefit from improved nutritional quality, particularly higher levels of vitamins and proteins [63].

Demand-driven efforts were therefore initiated to identify starch mutants within the germplasm collection. Native starches from 3272 landraces (including 12 wild relatives) and 772 improved clones were extracted and analyzed over several years. However, the recessive nature of these traits prevented the expression of phenotypes affecting starch functional properties, and the effort was therefore unsuccessful [61]. Subsequently, breeders undertook a systematic and meticulous process of self-pollinating accessions, growing the resulting S_1_ genotypes, and screening the extracted starch in the search of useful mutants. Several accessions carried mutations at the GBSS locus, responsible for amylose-free (waxy) starch [12,47]. These mutations were later introgressed into cassava breeding populations in Thailand [64].

Whiteflies represent a serious challenge to many crops, not only because of the direct damage caused by feeding but also—and perhaps more importantly—because they act as vectors of viral diseases, especially CMD and CFSD. *A. socialis*, *B. tabaci*, and *Aleurodicus dispersus* are among the species affecting cassava. Although whitefly resistance is rarely found in crops, cassava is a notable exception [48,49]. Such resistance has been identified in only a few landraces, most notably ECU 72, and involves both antibiosis and antixenosis. The trait appears to have a relatively simple genetic basis. A single QTL on chromosome 8 explains more than 35% of the phenotypic variation [45]. Additional QTLs have been identified on chromosomes 2, 5, and 14. This relatively simple inheritance is further supported by the successful introgression of resistance into the progeny of a cross between ECU72 × BRA12. Multiple years of field and consumer testing by both CIAT and Corpoica have led to the development and release of Nataima-3, a commercial variety with excellent levels of resistance to whiteflies [65].

Cassava brown streak disease (CBSD) was first reported in the 1930s in Tanzania. It remained largely a regional problem—also affecting Kenya and Mozambique—through the 1990s. However, over the past three decades, CBSD has expanded into several countries in Central sub-Saharan Africa [66]. Moderate levels of resistance have been gradually achieved in breeding populations [67]. This resistance is quantitative in nature and characterized by low-to-moderate narrow-sense heritability, making its transfer to new germplasm challenging. Recently, sources of strong resistance to CBSD were identified among 238 accessions tested from CIAT’s core collection (out of a total of 630 in the core) [50]. After two rounds of screening, 16 lines remained free of viral symptoms, and there was no virus detected [51]. Four resistance-associated markers explained between 8 and 33% of the phenotypic variance of CBSD severity or virus presence/absence [68].

The highly resistant accessions were from only three countries (Colombia, Ecuador, and Peru) out of a total of 20 countries that were sources of test material [50]. However, if tentative SNP duplicates are included [16], Brazil, Panama, and Venezuela can be added to the list. (Table S8 expands the description of sources of resistance to both CBSD and CMD to include tentative duplicates identified by Carvajal et al. [16].) This apparent concentration of resistance from Western South America provides a lead for follow-up on a possible basis for the origin of resistance in an area where the disease does not currently exist.

Cassava mosaic disease (CMD) has a long-term presence in Africa and India. Evidence for the early introgression of the resistance gene CMD2 into African breeding populations has already been reported. The introduction of the virus into Southeast Asia in 2015 provided a strong incentive to screen the germplasm for new sources of resistance [52]. Several accessions (CR63, PER262, TAI9, and VNM8) were found to carry the resistance gene CMD2. CR63 and TAI9 were likely duplicates based on SNP markers [16], while PER262 and VNM8 appeared to be unique genotypes among landraces in CIAT’s collection. Further, tentative duplicates of CR63/TAI9 appeared about 40 times—five countries in South America, four in Central America and the Caribbean, two in Southeast Asia, and one in the South Pacific [16]. This is a remarkable dispersal for a single landrace variety. We do not know whether the CMD2 gene may be linked with any other traits conferring some advantages in countries where the CMD virus does not exist. Of special note is the wide dispersal of CR63/TAI9 tentative duplicates in Indonesia. It is clear, therefore, that the New World germplasm contains multiple sources of resistance, some of which may have been subsequently introgressed into both African and Asian breeding populations where they were later identified [69].

The development of varieties with enhanced carotenoid content (biofortified), aimed at alleviating vitamin A deficiency, became a key breeding objective in the early-2000s [70]. Although most widely grown cassava varieties have white- or cream-colored parenchyma, germplasm with yellow-fleshed roots is not uncommon. Yellow-fleshed cassava and their associated high carotenoid levels are found even in regions distant from the crop’s center of origin [71].

Parenchyma pigmentation has long been associated with carotenoid content [72], and is largely controlled by the phytoene synthase (PSY) enzyme [43]. However, it soon became evident that there is considerable variation in carotenoid content among clones carrying the favorable PSY genotype. This indicates that additional genetic factors, beyond PSY, play an important role in carotenoid accumulation in roots. For example, downregulation of β-carotene hydroxylase has been shown to increase carotenoid accumulation in root and tuber tissues [73].

The cassava breeding project at CIAT implemented a rapid-cycling recurrent selection program focused exclusively on carotenoid content. Consistent and significant progress was achieved over a two-decade period, supporting the idea that carotenoid accumulation is controlled by more than a single gene and that multiple allelic variants likely exist at each locus. Several landraces from the germplasm collection—BRA1A, BRA1107, BRA 321, COL2489, COL2547, CR81, CR87, and PER297—were extensively used to introgress and exploit genetic variability in this program [53].

Adding other nutritional or palatability traits further complicates the adoption process and needs to be accompanied by significant marketing and demand creation. Consumers already use prior associations between observable traits, such as skin and flesh color, as well as culinary performance. It may be that biofortified cassava products have better initial penetration as a processed product (flour) than in the fresh consumption market. Additionally, parents used in this pipeline for biofortified cassava also need to include other essential performance traits to achieve a viable product with overall acceptable performance.

For non-biofortified varieties for direct consumption, cassava varieties have been developed for industrial uses—such as starch production, animal feed, or ethanol—are widely accepted. In contrast, improved varieties intended for direct household consumption, especially after boiling and without further processing, often lack consumers’ strict culinary qualities, which has significantly limited their adoption [4,74,75]. Recent studies have linked differences in cell wall compositions to variations in root cooking times [76,77], a key quality trait that can be screened quickly as a way to increase phenotyping throughput.

To address this gap, the RTBfoods project was initiated to promote the development of improved cassava varieties that aligned with consumer preferences for household consumption of cassava and other crops [78]. A central activity was the identification of several accessions—IND135, PER183, PER496, VEN77, MAL3, and COL1722—that exhibited excellent cooking quality and/or rapid softening upon boiling [26]. Ongoing research is expected to uncover additional sources for this important trait, which has strong implications for food security, particularly in Africa.

Post-harvest physiological deterioration (PPD) is a major obstacle in cassava storage, trading, and processing. Under ambient conditions, the storage life for retaining high-quality roots is typically only up to a few days after harvest. Tolerance to PPD has been a long-sought-after trait and would have huge economic benefits throughout cassava’s value chain. Considerable efforts have been made in understanding its biochemical nature [79] and the environmental/genetic factors influencing it [80]. For instance, increased carotenoid content reduces the severity of PPD. Unfortunately, there is a positive correlation between root dry matter content (DMC) and PPD.

For three decades, the cassava breeding project at CIAT has developed several populations and systematically evaluated them in order to select genotypes with commercially acceptable levels of DMC, extended shelf lives, and reduced PPD [54]. Tolerance has been introgressed from *M. walkerae* and a few other landraces from the germplasm collection (ARG 73, HMC1 (a hybrid developed from local landraces by ICA prior to CIAT’s foundation), and PER 182).

Natural tolerances to herbicides that inhibit the AHAS/ALS (Acetohydroxyacid Synthase/Acetolactate Synthase) and GS (Glutamine Synthetase) enzymes have been widely identified and exploited in various crops, including rice, wheat, maize, canola, sunflower, cotton, and quinoa. Several companies have developed proprietary brands associated with these tolerances, such as Clearfield^®^, STS^®^, Provisia™, SR Ready, LibertyLink^®^, and XtendFlex^®^. Because tolerance is linked to mutations at known loci (Table 9), gene editing has also been employed to develop tolerant varieties.

At CIAT, initial efforts focused on screening the cassava germplasm collection for spontaneous mutations in these loci using “molecular sieving” techniques, such as ECO-TILLING [56]. Herbicide tolerance is economically important for cassava, as it can potentially help it remain competitive with maize, a crop that shares several value chains. Equally important, herbicide tolerance can provide environmental benefits by enabling zero- or minimum-tillage practices, which reduce soil erosion and improve water-use efficiency [81].

Slowly digestible and resistant starches play an important role in mitigating type-2 diabetes and overweight-related health issues [82]. Starch digestibility is closely linked to amylose content [83]. High-amylose varieties have been developed in several crops through gene mutations in starch branching enzymes, such as SBEI and SBEII. In 2020, Zhou and colleagues [57] reported the development of transgenic cassava lines with suppressed expression of these genes. Downregulation of SBEII resulted in the amylose content exceeding 40%, confirming the role of this locus in controlling the trait in cassava. This finding opens the door for molecular approaches to identify accessions carrying spontaneous mutations at this locus.

Resistant starches are valued for their slow digestibility, but the opposite trait—rapid starch hydrolysis—also has important applications, particularly in the ethanol industry. Cassava is a major feedstock for ethanol production, especially in Southeast Asia [84]. A critical step in ethanol production from starchy raw materials is the breakdown of starch into shorter glucose polymers, which incurs costs in the form of energy or enzymes. Faster starch hydrolysis increases the efficiency of ethanol production.

### 8.3. Summary

The preceding information underscores CIAT’s efforts and commitment to identifying useful traits within the germplasm collection, and it enables us to draw several key conclusions and recommendations about the value of landrace varieties in discovering and harnessing rare, hidden, or unexplored but valuable traits, among other traits, to address challenges and opportunities that have arisen:

(1) Most of the traits sought in the germplasm collection were eventually identified.
(2) A wide range of traits can be detected through field evaluations in appropriate environments, but others only through specialized tools and methods.
(3) Several advanced laboratories possess extensive resources and infrastructure to conduct systematic screenings for useful traits, which could be uncovered using alternative “molecular sieving” approaches. Unfortunately, apart from the preliminary work by Duitama et al. [56], little effort has been made in this direction.
(4) Identification of useful traits is only the first step toward their exploitation. Many of these traits, often recessive, must be introgressed into appropriate genetic backgrounds and deployed through the release of improved varieties. In cassava, trait introgression remains cumbersome and inefficient due to the heterozygous nature of the progenitors typically used in breeding. For instance, the discovery of CBSD tolerance presents a major opportunity for cassava breeding in Africa. Over the past decade, substantial financial and human resources have been invested in implementing genomic selection in African cassava populations, gradually shifting allelic frequencies in the desired direction for most traits [85,86]. However, because these are closed populations, introgressing CBSD resistance would inevitably disrupt the allelic frequencies that have been painstakingly shifted over the last 15 years. A true breakthrough in cassava breeding could be achieved by moving breeding toward the use of partially or fully inbred progenitors (as discussed below).
(5) Other traits will continue to be added to this list. For example, field trials have identified a wide range of traits where genetic differences among a limited number of landraces have been noted, such as drought tolerance under well-watered versus rain-excluded plots [87], low versus normal levels of specific soil nutrients [88], and suitability for forage/silage [89]. These trials are costly to carry out and usually involve a very narrow range of landraces or breeding lines. We do not have enough information currently to speculate about the range of genetic diversity available. Nevertheless, the initial results from a limited number of accessions are promising.

## 9. Value of Landraces to Users of New Varieties

### 9.1. Released Varieties as the Foundation for Impact

In most countries, regulations stipulate that only officially released crop varieties should be promoted and supported. These released varieties are the foundation of eventual on-farm and consumer impact in most countries. While CIAT strongly supports the release process with its partners, it does not itself release varieties; this is a function solely at the discretion of and according to the processes and protocols of partner national agencies. Over 50 years, landraces and other accessions in CIAT’s cassava collection have participated in the pedigrees of well over 100 varieties released around the world (Table S9). A comprehensive compilation of these released varieties is complex and has not yet been fully developed. This partial list includes those materials with the best available information, and is based on: CIAT’s evidence of landrace status, selections from CIAT seed introductions, selections from local × CIAT crosses, and introductions by CIAT to national programs. Additional supporting evidence includes CIAT proceedings and other published manuscripts and reports.

An official release is not the only means by which varieties contribute value to users. For example, farmers may identify, obtain planting material, and successively evaluate breeding lines from experimental plots or on-farm trials. The authors have observed this phenomenon many times. The materials can become part of their named variety inventory [90].

Some countries do not have a well-structured release process for cassava and instead distribute materials more informally through an extension agency or by other means; this has been a common practice in the Caribbean region, for example [27]. The documentation of adoption and impact through these types of processes is sparse.

### 9.2. Farmer Adoption as the Crucial Test of Potential Value

While release is typically a precursor to impact in and of itself, release does not benefit end users of new varieties. They need to be adopted at scale and sustained over time with effective seed systems. The spread of new cassava varieties has been fastest outside the center of diversity in the Americas. In the case of SE Asia, expansive industrial demand, beginning in the 1970s, and the limited potential of local landraces to compete against non-cassava alternatives in global markets drove the development of successful breeding programs and rapid replacement of local landraces. In Thailand—Asia’s largest producer—nearly all the current cultivated area had already been planted with the products of breeding programs by the year 2000 [91].

There are no comprehensive global statistics on the adoption and impact of improved varieties of cassava. National program statistics, expert consultations, and farmer surveys, aided in some cases by DNA fingerprinting, have been used to track the progress of adoption and the identification of constraints.

Among the many countries with which CIAT exchanged germplasm, Thailand, Vietnam, Indonesia, Cambodia, Lao PDR, the Philippines, and China stand out in terms of the adoption of released varieties with introgressed Latin American diversity. Thailand has been the most careful in tracking the use of CIAT-derived materials in breeding (see for example [92]). The Kasetsart University team, led by Prof. Chariensuk Rojanaridpeched, selected KU50 from the combined genetics of local and introduced materials, including the landraces COL1505 and COL1684 in its pedigree. At its peak in 2009, the area planted with KU50 reached about 750,000 hectares in Thailand alone, and covered a total of nearly two million hectares across Asia [93].

Prior to the 2015 introduction of the cassava mosaic virus to Southeast Asia, improved cassava varieties derived in part from CIAT-related materials were adopted and grown on 2.7 million hectares across Vietnam, Thailand, Cambodia, Lao PDR, Myanmar, the Philippines, Indonesia, China, and India, representing 68% of the total hectares of cassava production across the nine countries [93]. Cambodia and Lao PDR experienced some of the world’s fastest growth in planted area in cassava production in the past two decades. However, both relied heavily on Thailand and Vietnam as sources of candidate varieties.

At the same time, as industrial varieties spread quickly and widely throughout SE Asia, local landraces continued to dominate the direct food market sectors. However, we are also beginning to get some insights into the New World sources of some of these landraces, well before breeding programs were established. For example, duplicates of the genebank accession CR63 [16] remain commonly grown throughout upland communities in Indonesia, where cassava is still an important source of food. The contributions of specific landraces from the crop’s center of origin, well before modern breeding programs, are starting to be revealed.

Colombia has released many varieties, with nearly all from the collaborative efforts with CIAT, including genebank sources (Table S9). ICA-Costeña was notable in that it was the first release from a fully farmer-participatory process in 1986–88. Most of the released varieties have had some success, but they have not heavily penetrated the very demanding fresh consumption (boiled roots) market that predominates in Colombia.

### 9.3. Value of Landraces Based on Economic Impact of New Varieties

As a comparative baseline, economists have estimated overall return on investment of the CGIAR system to be about 10% per year, with much of that attributed to improved varieties [94]. The value of crop genebanks as a component contributor has generally revolved around broad but unspecified contributions of genes for adaptation, productivity, resistance, quality, and resilience, which were often during the early history of breeding. However, nearly always, the focus of impact studies has been based only on yield differences between newer and older varieties (e.g., [95]). For cassava in Asia, both the total yield and percentage of starch play significant roles in varietal value, especially in terms of efficiency for starch extraction [91]. Most attributions of the economic benefits from CIAT’s genebank are based on accession participation in complex pedigrees. Separation of individual parental genomic contributions on a broad scale is hypothetically possible but has not yet been done. In this paper, we only suggest an overview and simplified approach to placing economic value on these benefits.

Thirty years ago, Henry [96] carried out a comprehensive projection on the expected benefits of CIAT-related cassava technology up to the year 2028, which is now nearly here. He noted that benefits from effective genetic resource management and genetic improvements are easiest to associate with CIAT activities because they provide concrete, physically identifiable technology components. Nevertheless, even this, as the easiest of variables (compared to crop management practices, for example), is highly complex to calculate, especially for the attribution of benefits from genebank accessions, as they make up only part of the complex genealogies of many new varieties. Henry predicted: “Genetically improved varieties will cover 35% of area in Asia, and 20% in Latin America and Africa thanks to a breeding history going back to the mid-1970s, and early results from the greatly expanded work in biotechnology of the early 1990s. Net benefits to technology adopters will average US$70/ha/yr from improved yield potential and quality (excluding pest/disease resistance, treated separately below), or about US$115m for Asia, $162m for Africa, and $41m for Latin America, in 1994 US dollars (assumes area increases of 30% in Africa, 20% in Asia and 10% in Latin America over 1993 levels)”.

In retrospect, these projections were very modest for Asia, where impact is best documented and CIAT’s genebank contributions are most accurately traced (Table 10). The growing demand for cassava-derived products has continued to drive the crop’s expansion in the past decade, with new areas almost exclusively dedicated to improved varieties for the industrial market segment.

In the case of S and SE Asia, we have calculated an approximate valuation of benefits using the figure of 2.7 million hectares grown using varieties derived in part from CIAT genebank sources (Table 11). The average yield has increased in Asia since CIAT began providing germplasm and technical support, averaging about 10 t/ha (Table 12). If we conservatively estimate just 10% of that increase to net annual benefits from traits contributed by genebank accessions and assume a modest US$50/t as a unit price of fresh roots, this equals $US135 million per year in net benefits. Given an operational cost of just a few million $US per year for CIAT’s cassava breeding and its related research, as well as cassava’s conservation in the genebank, this is a remarkable return on investment benefiting Asia’s farmers and its cassava industry that multiplies into the broader economy. Some of the key genebank accessions contributing to these benefits, based on their incorporation into widely adopted varieties, are COL22, COL1505, COL1684, MEX55, and VEN307.

Counterfactual to the success of the cassava breeding activities is that farmers would have continued to grow local landraces, while local breeding programs would still certainly have had some degree of success without the massive introduction of CIAT materials. However, this success would have been more modest, and the multi-billion dollars starch industry would not have evolved and expanded to the same degree without the innovation that delivered the high starch yield varieties—smallholder upland farmers would have needed to find alternative livelihood options. In the same way, the emerging diseases (CMD, CWBD) should not be viewed solely as an impact on yield but as a threat to the ongoing competitiveness and viability of the entire industry.

## 10. Roles of Landraces in an Inbred-Hybrid System: The Next Frontier for Cassava Breeding?

Cassava breeding has traditionally relied on the recurrent phenotypic selection of superior clones to largely exploit their additive genetic effects. However, evidence indicates that non-additive effects are substantial for key traits, such as fresh root yield and biomass. Earlier studies have shown that heterosis in cassava can be considerable, with root yield advantages ranging from 13% to over 136% relative to their parental lines [97]. Similarly, comprehensive analyses highlight that non-additive genetic variance is particularly important for complex traits like yield, while traits such as disease resistance tend to be more additive in nature. These findings underscore the opportunity to redesign cassava breeding programs to explicitly capture heterosis rather than relying on its incidental occurrence [98].

For cassava, “incidental heterosis” is routinely captured and maintained through vegetative conservation. This has made exploitation of heterosis in cassava a less compelling proposition compared to cross-pollinating with crops like maize, where incidental heterosis is lost in subsequent generations, and thus an inbred-hybrid system is required to stabilize it. Developing an inbred-hybrid system for cassava will be complex and long term, but there are compelling reasons to move forward with investment in its potential. In this section, we focus mainly on the specific roles that landrace varieties could play in an inbred-hybrid system, especially in the early years.

A central strategy for exploiting heterosis is the establishment of heterotic groups, which classify germplasm into genetically divergent pools that produce superior hybrids when crossed. This approach has been foundational in maize improvement, where reciprocal crosses between distinct heterotic groups consistently produce high-performing hybrids [5,99]. The same principle is applicable to cassava, where maximizing genetic divergence between parental lines increases the likelihood of favorable non-additive interactions. Advances in molecular markers and genomic tools can now allow breeders to define heterotic patterns in cassava populations more precisely, enabling systematic hybrid designs rather than empirical selections [46].

Heterotic groups/pools are genetically divergent populations that produce superior families or hybrids when crossed. An effective heterotic pool definition takes into consideration the genetic distance, the complementarity of allele frequencies among future pools, and the combining ability among the different potential parents. Such heterotic pools are not yet fully defined in cassava, making the foundational germplasm choice critical, particularly when it comes to sustaining longer-term genetic improvement [5].

Landraces have represented the building blocks for successful cassava variety development, and they will be central to any efforts aiming at exploring heterosis in cassava breeding. Cassava landrace accessions represent a foundational resource, offering genetic diversity, traits for adaptation and novel attributes, and evolutionary adaptative haplotypes essential for forming heterotic pools [100].

The use of landraces in building more effective breeding systems to exploit heterosis in cassava has its pros and cons. Cassava landraces are products of long-term selection under diverse agro-ecologies in farmers’ fields. This has led to broad general adaptation and resilience to predominant climate–soil conditions; complex trait combinations, including culinary and processing attributes; and resistance to locally predominant pests and diseases. Some of these attributes are often underrepresented in elite cassava lines, which are typically selected under a more narrow range of growing conditions [98].

From the genetic perspective, cassava landraces are characterized by high levels of heterozygosity and a high frequency of deleterious alleles, which are maintained through clonal propagation [5]. This, in turn, has positive implications for the exploitation of heterosis through complementarity of deleterious alleles and their positive allele counterparts [99]. However, such genetic features usually lead to severe inbreeding depression, which complicates the development of partially or fully inbred lines.

On the other hand, elite lines/varieties have demonstrated superior performance under specific growing/market conditions with known pedigrees and selection histories, as well as a relatively smaller load of deleterious alleles. As this review shows, a large percentage of the released varieties and elite lines trace back to a few landraces, meaning that, in general, elite germplasm would be suited for a short-to-medium-term hybrid development program with more predictable combining ability. The complementarity between cassava landraces and elite lines is shown in Table 13.

Genetic and genomic tools can aid in predicting and assembling heterotic pools that are most likely to maximize the expression of heterosis for performance traits, providing ample opportunity for allele complementation and the development of broadly adapted cassava hybrid genotypes [46]. Landraces preserve genetic distances that derive from their evolutionary backgrounds. When these landraces are admixed to create new hybrids, the heterotic character of those hybrids may not be maintained.

A practical example of heterosis exploitation in cassava is the breeding framework proposed and partially implemented by international programs, such as those led by CIAT and IITA. In this model, two broad germplasm pools, often representing distinct geographic or genetic backgrounds, are designated as heterotic groups A and B.

The breeding process could then proceed as follows:

(1) Diverse germplasm is grouped into two populations based on genetic distance, combining ability, and trait complementarity. Molecular markers (e.g., SNPs) are increasingly used to refine these groupings.
(2) Selected clones from population A are crossed with testers from population B, and vice versa. The resulting full-sib families or clones are evaluated in multi-environment trials for their yield, dry matter content, disease resistance, and quality traits.
(3) Hybrid progenies exhibiting strong heterosis (e.g., significantly higher root yield or starch content) are identified. Genotypes within those progenies can be selected, multiplied, and further evaluated as clonal varieties for potential release.
(4) The parents of superior hybrids are recombined within their original populations (A × A and B × B) to form improved breeding populations. Simultaneously, these selected parents can go through a round of inbreeding and inbred family selection aiming at building a pool of partially to highly inbred parents for future true-hybrid development.
(5) The process is repeated over multiple cycles, progressively enhancing the combining ability of each population and increasing hybrid performance.

This scheme mirrors the successful maize hybrid breeding paradigm but is adapted to cassava’s clonal propagation system. Importantly, once a superior hybrid clone is identified, it can be directly released and multiplied vegetatively, allowing farmers to benefit from the full expression of heterosis in the field [46].

The full realization of hybrid breeding in cassava faces several challenges. Producing homozygous lines through conventional selfing can take many years and may become difficult due to reduced plant vigor and low flowering; however, emerging technologies are beginning to address this limitation [37]. Partial inbreeding reduces the time required to develop progenitors and overcomes some of the problems related to inbreeding depression. The development and use of doubled haploids and inbred progenitors for cassava to create stable parental lines for hybrid production was proposed more than two decades ago [101]. These approaches aim to purge deleterious alleles and enable the systematic design of hybrids instead of relying on chance combinations. Such systems would represent a paradigm shift toward true-hybrid cassava breeding, analogous to maize. In parallel, genomic selection is being integrated into breeding pipelines to predict hybrid performance based on genome-wide marker data, significantly reducing the need for extensive field testing [102].

The road towards an inbred-hybrid cassava breeding system will be likely based on an integrative strategy, where elite germplasm could drive early hybrid success, but landraces will be used to build and diversify heterotic pools, with a strong application of genomic tools to bridge the two efficiently and enable the development of inbred parents with superior combining ability.

## 11. Projections for the Further Exploration and Use of Landrace Varieties

A historical review of the cassava germplasm collection provides a basis to articulate the lessons learned and provide guidance on how to better prepare for the future. A basic way to summarize our understanding about inherent diversity of landrace traits might be the following: “if you can measure any trait precisely, and statistically separate out environmental variation and random error, you will likely discover some degree of genetic variation”. This is the legacy of 50 years of evaluation of both breeding lines and landrace varieties. Nearly every imaginable trait measured shows some degree of genetic diversity. Whether the range of variation is sufficient to be useful, or whether there is an economically viable pathway to warrant efforts in breeding, are separate calculations. At the same time, CIAT has always recognized that not all challenges or opportunities are best approached by breeding, or even by breeding alone. Breeding is fundamentally informed by a fully multidisciplinary effort, which includes agronomy, soil science, physiology, entomology, plant pathology, utilization, economics, the social sciences, and others.

Pardey and Beintema [103] described agricultural research and development as “slow magic”. They recognized the extraordinary successes of the CGIAR system in helping those in lower economic strata by leveraging focused technological benefits. This is especially exemplified by the creation of new crop varieties that have improved the livelihoods of billions. The great majority of benefits created by the CGIAR system resulted from steady progress over decades of effort—from the inception of research to the results impacting people’s livelihoods.

The genetic diversity provided by landraces and other related species are a key part of the foundation on which this strategy is built. Long-term, comprehensive breeding programs that integrate the best germplasm and the best tools (e.g., genomics, high-throughput phenotyping, and thorough market testing) are essential for driving accelerated progress. A better understanding of producer and consumer needs is getting more attention as fundamental to equitably benefiting women, lower income strata, and other priority groups. Climate change and concerns about environmental degradation play strong roles in current research priorities. All these factors are dynamic; therefore, it is imperative that breeding programs are forward-looking, with the knowledge that agricultural and food systems are undergoing rapid transformation.

After 50 years of evaluating cassava landraces and extracting their benefits, the potential for “quick wins” from the genebank may seem to be remote. A newly discovered landrace variety that could be directly recommended for broad adoption or a new high-impact trait for quick incorporation into advanced breeding lines are low-likelihood scenarios. The genebank has always been a long-term concept, one of conservation, legacy, and leverage. It will continue to provide value to breeding programs but with a necessarily long-term mindset for planning and funding.

With the above caveats, advances in plant breeding tools and in efficiencies for using landraces offer glimpses of pathways for accelerated gains. These gains are dependent on increasing investment in cutting-edge genetic tools and quick deployment of those tools, which are then fully integrated into well-supported pipeline-based breeding programs that include high-throughput phenotyping systems. At the end of the process, clean and viable planting material must be systematically transferred to farmers.

The following list of potential emerging challenges and opportunities is not comprehensive, but it is intended to inform in a broad way about the critical need for long-term planning that may draw on the potential contributions of landrace varieties.

### 11.1. Continuing Vulnerabilities from New or Shifting Pest and Disease Pressures

Breeders have been highly successful in attacking several major pests and diseases through natural resistance, as well as plant protection specialists through biological and crop management practices. However, history tells us that pests and diseases will be a perpetual, ongoing challenge. We are half a century past the common belief that cassava, as a species, was somehow highly resilient to pests and diseases. While many pests and pathogens have remained localized in either the Americas or Africa, their intercontinental introduction is a continuing threat. Likewise, even where some pests or diseases have long been present but at low levels, environmental and production system changes can cause increases in severity.

Neither cassava mosaic disease nor cassava brown streak disease have been reported in the Americas, nor cassava frogskin disease in Africa or Asia. Every precaution needs to be taken to be prepared for these eventualities, including building preemptive resistance in breeding lines.

The rapid spread of cassava mosaic disease (CMD) after its first reported incidence in 2015 [104], and the increasing importance of cassava witches broom disease (CWBD) by the mid-2020s, are now driving a massive replacement of some of the older-bred industrial varieties that lack resistance to these diseases in mainland SE Asia. Yield potential and higher starch content alone will not be the sole drivers of adoption. In addition, many of the persistent landraces of SE Asia that remain an important component of food security may face a similar fate in the face of disease. Interdisciplinary teams from multiple partner institutions are joining forces to bring new genetics into the breeding pipeline for different market sectors, with the task of stacking traits that meet farmer, processor, and consumer expectations. The landraces from CIAT’s genebank and from the IITA have demonstrated efficacy for CMD, and the search is underway for CWBD resistance.

### 11.2. Impact of Global Trends in Production and Use

At the inauguration of CIAT and IITA in the late-1960s, Africa already accounted for 56% of cassava area planted at a global level, and this climbed rapidly to 80% by 2022. While demand was high, the yields were tenaciously level when compared to Asia, where productivity per unit area doubled in the same period. This demand in Africa was met by significant planting area expansion. But even in Africa, where land is under far less pressure than in Asia, the reliance on area expansion is not a sustainable long-term solution for food production.

This lag in productivity improvement in Africa ostensibly caused a major shift in funding for cassava research. Multilateral donor support has moved aggressively toward Africa-related research over the past three decades while declining for Asia and Latin America. Nevertheless, several countries from the latter two regions adjusted to this changing research paradigm by increasing, or at least maintaining, internal investments and building networks that reinforced collaboration. Brazil is certainly the main investor in the Americas, reasonably accounting for about 60–70% of total production in the Americas over the past 50 years. Compared to Asia, Brazil has far greater access to local genetic diversity. In this environment, it is essential to continue to recognize the cassava collection of landraces as a critical global resource.

### 11.3. Climate Change and the Move into New Production Areas

Climate change will increasingly challenge farmers and the research systems that support them. There are going to be new demands on cassava varieties and thus on CIAT’s genebank and the variety development process. A typical response from donors and public research organizations (including CGIAR) is to support germplasm screening and breeding for drought and high-temperature tolerance. Yet the lessons learned from the first fifty years of landrace evaluations and their use indicate that a more nuanced and integrated response is probably needed for cassava.

One early review of the evidence suggested that the main threats to cassava from climate change will be from shifts in pest and disease pressures [105]. Though it may seem counterintuitive, the evidence would suggest that “in areas where drought stress is likely to become more prevalent, research should focus on secondary results rather than on physiological responses to drought itself”. Breeding and crop management strategies that balance pest and disease resistance with heat and drought tolerance will be critical strategic options. The mechanisms for drought tolerance and photosynthetic efficiency, at the physiological or morphological levels, are potentially strong candidates in the search for genetic diversity among landraces.

Managing quality traits under heat and drought stress, especially for the capacity of a variety to maintain or recover high root starch content up to harvest time, will also draw on the genetic diversity of both advanced breeding lines and of landraces. Because of the long-growing period, this trait is fraught with multiple complexities in measurement and is a target for improvement. While the focus is often on drought and heat tolerance, more frequent and intensive rainfall events are resulting in waterlogging, leading to root rot and low starch stability in some of the main cassava-producing regions of Asia.

### 11.4. Advances in Mechanization of Production and Harvest Systems

Across its many components of production and post-harvest management, cassava is a challenging crop to mechanize. However, mechanization will inevitably advance in order for production systems to remain competitive and support better livelihoods. Labor productivity increasingly drives the viability of agriculture, and mechanization is a primary method to achieve this goal. This is not to say that the traditional labor-intensive systems of past centuries will disappear from production systems any time soon, but that their comparative global importance will continue to decline. To date, the story of cassava mechanization has largely been one of mechanical engineers adapting machinery to cassava’s innate traits rather than plant breeders adapting cassava to better fit options for mechanization. There may be ways, through exploiting genetic variation, to better integrate these two approaches. For most planting management and harvest systems, it seems unlikely that breeders will need to go beyond the genetic diversity available in advanced breeding lines. Nevertheless, some specialized traits may require a search among landraces.

### 11.5. Processing Operations

Several phases of post-harvest processing are easily mechanized without much complication. One very significant exception to this generalization is peeling. Most uses of cassava, especially for human food, require removing the peel. Peeling has been mechanized, but the available technologies tend to produce excessive waste (i.e., remove more of the root flesh along with the peel, as compared to hand peeling) and leave some peel attached at the end of the process. For nearly all food uses, peeling is still done manually, and mostly by women. Issues of gender-responsive technology are highly relevant both to ease manual peeling and to facilitate the mechanization of peeling. There are several root characteristics that impact peeling:

(1) Root size.
(2) Undulations/unevenness of the root surface.
(3) Strength of adherence of the peel to the root parenchyma (flesh).
(4) Peel thickness.

These are all genetically complex factors, and as of now they are poorly understood from a breeding perspective. Root peel traits, such as strength of peel adherence and peel thickness, are areas for possible landrace screening in the search of new genetic variation to optimize efficiency of both manual and mechanical peeling.

### 11.6. Traits Better Oriented to the Needs of Urban Markets

Urbanization impacts trends in market demand. Broad-scope drivers of change for cassava include: demand for convenience products (storability, ease of preparation); cost competitiveness; nutritional value; and food safety. On the other hand, recent generations of migrants from rural to urban settings, or some niche markets, now seek traditional products such as fresh cassava for consumption after boiling.

These represent a complex set of product demands that require a combination of management (processing, transportation, storage) and genetic solutions. In-depth research into the details of consumer demand for cassava under evolving rural–urban dynamics is relevant globally. Traits related to starch functionality, post-harvest deterioration, and secondary metabolites, among others, would benefit from further exploration of diversity in the genebank.

The potential for plant-derived biologicals and pharmaceuticals is nearly limitless—for nutrition, health, flavorings, cosmetics, etc. This will likely be an industry-driven search, but it could also be of public interest, especially in the areas of nutrition and health.

Specialty starches (beyond the amylose-free mutant discovered by CIAT and already in commercial use) are frequently of interest to industry. The consumer trend toward natural products favors unmodified starches rather than chemical modification or genetic engineering. Similarly to the waxy starch experience, the demand for and discovery of new specialty starches will benefit from a search among landrace varieties.

## 12. Summary and Conclusions

CIAT holds in trust the world’s most extensive collection of landrace varieties, mainly from the crop’s center of origin. The collection, first established in 1969–1970, currently consists of about 5000 landrace accessions, along with representatives of 23 wild *Manihot* species and elite hybrids from CIAT and its partner breeding programs. CIAT established a breeding program in 1972, with the first goals of evaluating the collection and developing a pathway toward its use for creating new varieties. After just two years into that process, CIAT’s breeders concluded that few landrace varieties could directly meet future needs in a world where higher yields and better nutrition were demanded. Attention turned from germplasm evaluation to making crosses to combine desired traits. A preliminary selection based primarily on root yields and harvest indices (the proportion of root yield to total yield) provided a base of 230 accessions that would dominate the parental pool, as well as any subsequently derived hybrids, for several decades.

From the outset, CIAT directed considerable resources toward supporting both the few established breeding programs and the many newly emerging ones in Asia and the Americas (while IITA focused on Africa, according to the CGIAR mandates). This included the exchange of seeds from crosses made at CIAT, mainly among the 230 initially selected accessions. The rapid decentralization of breeding efforts and local selection by partners were critical for breeding success. This success moved especially quickly in SE Asia, where the main goals were to increase yield potential and starch content for industrial use—initially as dried chips for export to Europe for the cattle feed industry, and then as starch.

In the Americas, the challenges were more diverse and complex. By the late-1970s, the headquarters cassava team concluded that there was a need to revisit and reevaluate the collection under a wide range of environments. The diversity of agro-ecosystems and market needs—with a range of biotic and abiotic constraints and required quality traits for different products—required a subdivision of breeding objectives. This involved the definition of seven “edapho-climatic zones” (ECZs). Among these, only the subtropical zone was not represented in Colombia. This initiative involved a massive new evaluation of the germplasm collection in several sites around Colombia. Accessions with the best performance (generally combining critical adaptation, yield, resistance, and quality traits) were combined within ECZ-oriented gene pools, followed by successive generations of recurrent selection. This revisit of the genebank resulted in the identification and use in breeding of a few hundred additional accessions as part of the breeding pool. This history places cassava, among other major crops, as one that has made the most use of broad-based genetic resources in breeding. The collection has contributed to the pedigrees of over 100 released varieties around the world, mainly in Asia and the Americas, but also with a few in Africa.

Systematic field-based phenotyping in a recurrent selection scheme has been highly successful when measured by adoption and impact, especially in Asia. But new challenges, along with new information, tools, and institutional capacity, are shifting the ways in which the genebank is perceived and utilized. Broad-based improvement of agro-ecosystem adaptation, yield potential, target market quality, and host plant resistance are on-going with national programs. CIAT will continue to explore landrace diversity toward more specific targets. An earlier example, the discovery of a single gene controlling for amylose-free starch, was a game-changer in this pathway. A decade ago, the introduction of cassava mosaic disease (CMD) to SE Asia drove the mobilization of joint efforts by CIAT and IITA in the search for resistance in adapted backgrounds. Among the revelations was the discovery not only of CMD resistance but also of resistance to the cassava brown streak disease among Latin American landraces, neither of which had been reported in the Americas. Other diseases are creating new or expanded threats, such as cassava witches broom disease and cassava frogskin disease, and the search for resistance is ongoing. The history of agriculture tells us that there will be a continual evolution of pest and disease challenges long into the future. Thus the genebank will always be a first line of investigation.

Cassava is still a crop that is highly labor-intensive and difficult to mechanize. But as countries develop, labor efficiency becomes increasingly critical. Cassava breeders will increasingly need to offer traits that facilitate mechanical planting (long, straight stems), mechanical harvest (more compact roots), longer storage time in the marketing process, and easier peeling (root form and peel characteristics). Some of these traits are already readily accessible in advanced hybrids, whereas others will rely on germplasm screening.

Cassava breeding, as a science, faces the dual challenges of accelerating progress in breeding and historical underfunding, with almost no private sector involvement. Breeding is traditionally slow due to long growing seasons (i.e., slow breeding cycle), the heterozygous nature of all varieties leading to unpredictable segregation, and irregular flowering. After many years of breeders dreaming of working with an inbred-hybrid system for high-precision breeding, this approach is finally getting off the ground. It will eventually allow for the much more efficient extraction and use of traits found in less-adapted germplasm, as well as the application of genomic tools to accelerate selection. In all these challenges/opportunities, landraces have a key role to play. We have an immense range of cassava genetic diversity available today as a result of the cultivation and selection skills of the ancestors of today’s farmers who generously contributed their varieties to the genebank.

## Figures and Tables

**Table 1:** Trends in the R&D world impacting priorities for cassava germplasm evaluation and breeding ^a^.

Approximate Period of Time	Broad Trends in R&D Strategic Targets Across Development Organizations	Examples of Prioritization by CIAT Cassava Breeding Team for Use of the Germplasm Collection
1967–1972	Protein deficiency as the main global nutrition concern	Protein content and quality (before beginning a formal breeding program)
1973–1980	The Green Revolution’s emphasis on high yield under well-managed conditions	Efficient yield, based on medium-to-high inputs + high yield potential + high root dry matter + high harvest index
1981–2000	Focus on cassava’s comparative advantages in difficult growing conditions, within specifically defined edapho-climatic zones	Moderate-to-high yield under regionally prevalent biotic and abiotic stresses, with a focus on genetic resistance/adaptation
2001–2010	Specialty traits for niche markets	Amylose-free (waxy), especially for industrial use
2011–2020	Biofortification for human nutrition	Beta-carotene content for regions with vitamin A deficiency
2021–present	Emerging pests and diseases, shifting consumer preferences, and increasing climate extremes	High, stable starch yield; erect, mechanization-ready architecture; consumer preference for fresh-market quality; resistance to cassava mosaic and brown streak virus(es); tolerance to witches broom; and early maturity/root bulking for flexible harvest under extreme weather

^a^ Classification by authors.

**Table 2:** Characteristics of CIAT germplasm accessions used frequently in early hybridizations, with predominant traits based on first agronomic evaluations ^a^.

Accession	Origin	Harvest Index	Total Plant wt.	Leaf Area Retention	Early Maturity	Ease of Harvest	Cassava Bacterial Blight Resistance	Superelongation Disease Resistance	No. of Cross Combinations ^b^
COL22	Lowland semi-humid	++			++	++			128
COL113	Mid-altitude			++					66
COL197	Highland						++		1
COL647	Acid soil savanna						++		46
COL667	Acid soil savanna						++		9
COL755	Acid soil savanna	+	+	++					22
COL914	Lowland semi-humid	++			+				69
COL1438	Acid soil savanna	+					+	+	75
MEX23	Acid soil savanna		+	+			+	+	1
MEX55	Acid soil savanna	++		+	++				41
MEX59	Lowland humid	+	++	+					14
VEN185	Lowland semi-humid	++	+						76
VEN270	Unknown	+	+						56
VEN307	Unknown	+	+						12

Source: Ref. [14] and additional information from https://www.cassavabase.org (accessed 15 June 2026). ^a^ + = good for a trait; ++ = very good for a trait. ^b^ Number of cross combinations in which an accession appears, either as one of two parents in a controlled cross, or as the female parent for open pollination.

**Table 3:** Examples showing the quickly narrowing number of genebank accessions, from a starting population of 3028, that meet multiple additive criteria for overall good performance in three agroclimatically contrasting regions of Colombia.

Target Area/Test Site	Basic Breeding Objectives	Target Level of Expression	Accessions Meeting Criteria
Subhumid Tropics: Colombia’s Northern Coast			
Root yield (kg/plant)		>3.5	1007
Plus harvest index		>0.5	432
Plus root dry matter content (%)		>35	313
Plus cassava green mite damage ^a^		<2.0	57
Plus thrips (*Frankliniella williamsi*) damage ^a^		<2.7	42
Acid Soil Savanna: Carimagua, Meta Dept.			
Bacterial blight damage ^a^		<3.0	55
Plus superelongation disease damage ^a^		<3.0	21
Plus cassava green mite damage ^a^		<3.0	6
Plus lacebug (*Vatiga illudens*) damage ^a^		<3.0	4
Highlands: Popayan, Cauca Dept.			
Cool temperature tolerance and concentric ring leaf blight (*Phyllosticta manihotae*) damage ^a^		<2.0	17
Plus red spider mite (*Tetranychus* spp.) damage ^a^		<2.0	10

Source: Ref. [9]. ^a^ Ratings on a 1-to-5 scale, where 1 = very low damage and 5 = very high damage.

**Table 4:** Descriptions of edapho-climatic zones defined by CIAT’s cassava program as broad guidelines for evaluating germplasm and for gene pool development.

No.	Description	Representative Countries/Regions	Principal Constraints ^a^
1	Subhumid lowland tropics	NE Brazil; Colombia (Atlantic coast; Santanderes); N. Venezuela; Mexico (Yucatan Peninsula); most cassava-growing areas of SE Asia and East Africa; East Java; subhumid belt of sub-Sahelian Africa; South India	Drought stress; mites; thrips; *Diplodia* and *Fusarium* root rots; mealybug; cassava mosaic disease; cassava brown streak disease; whiteflies
2	Acid soil, lowland tropical savanna	Brazil (Cerrado); Colombia (eastern plains); Philippines; W. African savannas	Soil acidity; bacterial blight; superlongation disease; anthracnose; mites; mealybug; cassava mosaic disease; whiteflies
3	Humid lowland tropics	Amazon basin (Brazil, Colombia, Peru); West Java and Sumatra; Malaysia; Southern Vietnam; equatorial West Africa	*Phytophthera* and *Fusarium* root rots; cassava mosaic disease; anthracnose; *Cercospora* and *Cercosporidium* spp.; mealybug; whiteflies
4	Mid-altitude tropics (800–1400 masl)	Andean zone; Central Brazilian highlands; Jos plateau of Nigeria; Cameroon; East Africa	Thrips; mites; root rots; mealybug; cassava frogskin disease; anthracnose
5	High-altitude tropics (>1400–2200 masl)	Andean zone; Rwanda; Burundi	Concentric ring leaf spot; low temperature; bacterial blight; anthracnose
6	Subtropics	Southern Brazil; Northern Argentina; Paraguay; Cuba; Southern China; Northern Vietnam; Southern Africa	Low winter temperature; bacterial blight; superelongation disease; *Cercospora* and *Cercosporidium* spp.
7	Semi-arid lowland tropics	Northeast Brazil; Guajira of Colombia; semi-arid belt of West Africa; UR Tanzania; Mozambique; lowlands of Rwanda and Burundi	Drought stress; mealybugs; mites

Source: Adapted from [9]. ^a^ Not all constraints are found in all regions of a given edapho-climatic zone—common name (scientific name): mites (mainly *Mononychellus* and *Tetranychus* sp.); thrips (*Frankliniella williamsi*); cassava bacterial blight (*Xanthomonas axonopodis* pv. *Manihotis*); superelongation disease (*Sphaceloma manihoticola*); concentric ring leaf spots (*Phoma* spp. syn. *Phyllosticta manihotae*); cassava mealybugs (*Phenacoccus herrenii* (most of the Americas) or *P. manihotae* (Africa)); cassava whiteflies (*Aleurotrachelus socialis* (Latin America) or *Bemisia tabaci* (Africa)); cassava witches broom disease (*Ceratobasisdium theobromae*); anthracnose disease (*Colletotrichum gloeosporioides* s.l.).

**Table 5:** Summary of genebank evaluations in diverse environments of Colombia from 1980 to 2023.

Zone Description	Evaluation Site	No. of Trials	Genebank Accessions Evaluated ^a^
Lowland seasonally dry (ECZ 1)			
	EL CARMEN, BOLIVAR	10	294
	EL ESPINAL, TOLIMA	1	27
	MEDIA LUNA, MAGDALENA	40	4666
	SANTO TOMAS, ATLANTICO	7	1835
	TAMALAMEQUE, CESAR	1	511
	VALLEDUPAR, CESAR	5	259
	Total ECZ 1	64	7592
Lowland acid soil savanna (ECZ 2)			
	CARIMAGUA, META	16	1976
	LA LIBERTAD, META	13	2268
	Total ECZ 2	29	4244
Humid rainforest (ECZ 3)			
	BUENAVENTURA, VALLE	1	22
	FLORENCIA, CAQUETA	9	1943
	Total ECZ 3	10	1965
Mid-altitude inter-Andean (ECZ 4)			
	CANDELARIA, VALLE	1	22
	CIAT-PALMIRA, VALLE	56	7277
	PATIA, CAUCA	1	31
	QUILICHAO, CAUCA	1	48
	Total ECZ 4	59	7378
Highland inter-Andean (ECZ 5)			
	CAJIBIO, CAUCA	3	387
	POPAYAN, CAUCA	9	2394
	Total ECZ 5	12	2781
Semi-arid (ECZ 7)			
	AREMASAIN, GUAJIRA	2	129
	Total ECZ 7	2	129
	TOTAL	176	24,089

Source: https://www.cassavabase.org (accessed on 12 February 2026) and unpublished CIAT cassava program data. ^a^ Most accessions were evaluated across multiple sites, and the totals include repeated evaluations. These totals do not include trials in which 20 or fewer accessions were evaluated. Trials with only a few included accessions were typically not designed for the purpose of evaluation of the germplasm collection per se.

**Table 6:** Genebank accessions included in the pedigrees of 50 or more hybrid combinations by CIAT’s cassava breeding program up to 2000 during the era of edapho-climatic zone-oriented breeding.

Accession	Local/Other Names (Dept. of Collection)	Used in No. of Crosses	Key Traits Contributed in Crosses ^a^
BRA12	BGM 1008; BRA-077950	266	Mite and insect resistance
COL1505	UCV2096; Bra 1280	241	Tolerance to pests and diseases; eating quality; widely adapted
COL1468	Mantiqueira; Sip24; Bra-000965; CMC40	233	High yield and disease tolerance; low DM; pest susc.; widely adapted
TAI8	Rayong 60	174	High yield and high DM in SE Asia
COL638	Chiros/Yema de Huevo (Meta)	158	Resistance to diseases; moderate carotenoids
BRA5	(No information)	156	
NGA2	TMS30572	131	Resistance to CMD
TAI1	Rayong 1	131	Wide adaptation
COL22	Uvita (Cordoba)	128	Short stature; high dry matter
COL2215	Venezolana (Magdalena)	127	High dry matter; eating quality
PAN114	Papito (Veraguas)	119	Good yield and DM; wide adaptation
COL1522	Algodonera amarilla (Cauca)	108	Adaptation to high altitudes
VEN77	UCV 2180; Mcol 1914; CMC 210	80	Resistance to CBB and SED
COL72	Ramirana (Sucre)	79	Short stature; high dry matter
MEX1	No name (Veracruz)	76	Wide adaptation
VEN185	Morada (Barinas)	76	Wide adaptation
COL948C	Algodon (Cordoba)	75	
COL1438	Llanera; CMC 9	75	Resistance to diseases; moderate carotenoids
COL1684	Charay; CMC 163 (Amazonas)	74	High harvest index; low DM; tolerance to diseases
NGA1	TMS 30001	74	Resistance to CMD
COL1495	Santa Catarina (Caldas)	73	
COL914	Venezolana (Bolivar)	69	
COL2207	Enanita (Cesar)	67	Short stature; high DM
COL113	Negra (Valle del Cauca)	66	High vigor; good leaf retention
COL1823	Tempranera (Arauca)	64	Wide adaptation
COL1413	Cacota (Santander del Sur)	59	
NGA5	TMS 30555	57	Resistance to CMD
COL1805	Botoncito (Magdalena)	56	Wide adaptation
COL1910	UCV 2141; CMC 206;	56	
MAL2	Black Twig	56	Popular local variety in Malaysia
VEN270	(No information)	56	
COL10	Mona Blanca (Cordoba)	51	Short stature; good DM
CUB74	Señorita	51	Good eating quality
COL976	Secundina (Atlantico)	50	Good eating quality
COL2060	Regional Amarilla (Cauca)	50	Adaptation to high altitudes

Source: https://www.cassavabase.org (accessed on 25 June 2026) and unpublished CIAT cassava program data, compiled by N. Morante, CIAT. ^a^ DM = dry matter; CBB = cassava bacterial blight; SED = superelongation disease; CMD = cassava mosaic disease.

**Table 7:** Historical basis for genebank accessions in pedigrees of CIAT cassava crossing nursery parents, leading to the current (2021–2025) use in priority pipelines for product development for key market segments ^a^.

	Historical Basis for Selection		2021–2025 Priority Pipelines					
			Participating in Key Target Markets				No. of Families Prod.	No. of Seeds Prod.
Code	Selec. in 1973–74 at CIAT ^b^	Most Used in ECZ Selection ^c^	Biofortified	Cooking Quality	Processing	Low Amylose ^a^		
ARG73				X	X	X	29	1979
BRA1016				X			21	300
BRA12		X			X	X	9	85
BRA1428				X	X		3	32
BRA191		X		X	X		1	138
BRA334			X	X			14	819
BRA384				X	X		3	194
BRA787		X		X			31	878
BRA847				X			8	361
BRA97		X		X			6	87
COL144		X	X	X	X		48	17,543
COL1468	X	X		X			18	496
COL1505		X	X	X			48	4838
COL1516		X	X	X		X	36	1034
COL1522		X			X	X	11	184
COL1566				X			21	459
COL1667				X			1	2
COL1684	X	X			X		11	806
COL1722		X		X			1	262
COL1734		X		X	X		16	1485
COL1736				X	X	X	8	289
COL1764A				X	X		6	130
COL1793		X			X		17	557
COL1819				X	X		2	4
COL2182			X	X	X		41	856
COL22	X	X			X		3	28
COL2204				X			9	89
COL2215		X	X	X			55	3432
COL2246				X	X		12	214
COL2253		X			X		12	512
COL2564				X			4	68
COL2627				X			1	348
COL2714				X	X		6	56
COL2728				X	X		2	9
COL32	X			X			18	531
COL40		X	X	X	X		25	10,375
COL638	X	X			X		9	168
COL65	X	X		X	X		2	5
COL71				X	X		7	47
COL764		X		X			28	1650
COL982A	X			X	X		3	14
CR63		X	X	X	X		45	2319
CUB46		X		X			22	749
CUB74		X		X			35	1294
ECU41			X	X	X		3	667
ECU72		X		X	X		11	261
IND135				X			8	130
MAL3		X		X			18	3437
MEX1		X			X		3	62
MEX2		X	X	X			21	588
MEX55	X	X		X	X		1	82
MEX59	X				X		2	12
MEX6				X			1	10
NGA11			X	X	X	X	57	1777
NGA19		X			X		5	183
PAN70		X	X	X			11	261
PAR68				X			7	124
PER183		X		X			51	4317
PER221				X	X		91	11,577
PER328				X			34	1295
PER333			X	X			12	94
PER347				X			5	278
PER368				X			5	64
PER415					X		5	138
PER496				X			9	159
PER556				X	X		19	153
PER608				X	X		3	790
PTR19		X		X			2	10
TAI1		X			X		1	110
TAI27					X		1	68
AI5		X			X		3	52
TAI8		X			X	X	17	3197
VEN218	X	X		X	X		1	6
VEN25		X			X		9	364
VEN270	X	X		X	X		2	209
VEN77	X	X		X			16	489
Partic. clones	12	39	13	60	43	7	Total	86,690

Source: CIAT cassava program’s unpublished data. ^a^ X indicates participation of a clone in a breeding pipeline for key target markets. Many clones in the genealogies are the result of open pollination, and thus with an unknown male parent. Therefore, the genetic base of these pipelines will be somewhat wider than indicated by this table. ^b^ Accessions included in the first 230 selections at CIAT headquarters in 1973–74. ^c^ Based on accessions participating in hybridizations for superior performance in Colombia’s diverse edapho-climatic zones (ECZs).

**Table 8:** Description of categories of traits to address arising challenges and opportunities, with historical and current examples.

Description	Examples
Low frequency: Traits that appear to be expressed at a low or very low frequency in the collection, and may be expressed across a range from low to high levels when they do appear.	Resistance to whiteflies, cassava mosaic disease, and cassava brown streak disease
Hidden due to recessive-dependent expression: Traits expressed only in a homozygous recessive state, and are therefore “hidden” until homozygotes are produced through inbreeding or revealed through molecular markers.	Amylose-free (“waxy”) starch
Challenging to measure expression: Appropriate techniques are not yet developed or not yet adapted to cassava; in some cases, due to very high GxE interaction. The presence/absence, frequency, or level of expression are often unknown due to the difficulty of measurements.	Resistance to cassava frogskin disease or cassava mealybug; post-harvest deterioration
Unknown nature of expression due to lack of prior exploration: Traits that have not yet come under the radar of user interest.	Numerous traits related to physiological processes, starch functional properties, and reproductive variations

**Table 9:** Examples of traits addressing arising challenges and opportunities.

Trait	Report	Key Accessions	Utilization
Amylose- free (“waxy“) starch GBSS	[47]	Not publicly disclosed ^a^	Introduced to Thailand and Brazil; extensive crossing for selecting improved varieties in Colombia (public–private partnership).
Resistance to whiteflies	[48,49]	ECU72	- Resistant variety released in Colombia (Bra 12 × Ecu 72). - Introduced to Africa through IITA. - Broad array of crosses to incorporate the trait for multiple target regions.
Resistance to CBSD	[50]	16 accessions, incl.: COL40, COL144, COL2182, ECU41, PER315, PER353, PER556,	- Crossed with locally adapted susceptible types in Africa. - Incorporated into Latin American and Asian varieties as a preemptive strategy in the case of pathogen introduction. - Explored as a means of transfer to susceptible types via gene editing.
Resistance to CMD	[51,52]	CR63 ^b^	Initially tested in Vietnam to combat the CMD threat in SE Asia. Replaced by higher starch varieties as sources, especially CR52A-4 ^c^ [52].
High carotenoids content	[53]	BRA1A, BRA1107, BRA1321, COL2489, COL2547, CR81, CR87, PER297	Further increase the levels of carotenoids among yellow-fleshed cassava genotypes [53].
Boiling quality	[26]	IND135, PER183, PER496, VEN77, MAL3, COL1722	Introgressed into African breeding populations for cooking quality traits, for which there is no wide variability in Africa [26].
Tolerance to PPD	[54]	ARG73, PER183, HMC1	First germplasm with good root dry matter content, white parenchyma, and tolerance to PPD [54].
Tolerance to glyphosate	[55]	Ten clones highly tolerant, including ECU81 and Rayong 5 (MEX55 and VEN307 in pedigree)	Tentative tolerance to a range of doses of glyphosate sprayed on potted plants of 262 genotypes; requires follow-up confirmation [55].
Tolerance to herbicides inhibiting AHAS or ALS (mutations at Pro197, Trp574, or Ala122 loci)	[56]	None identified	Tolerance to imidazolinones, sulfonylureas, pyrimidinyl-thiobenzoates, sulphonyl-aminocarbonyl- triazolinone, and triazolopyrimidine (e.g., Clearfield^®^, STS^®^, Provisia™, SR ready) [56].
Bialaphos tolerance (“BAR” or “PAT” gene)			Tolerance to glufosinate herbicides (e.g., Basta, Rely, Finale, Liberty, etc.).
Amylose extender SBEII	[57]		Resistant starches desirable for health concerns, particularly for diabetes type-2 and overweight [57].
Fast starch hydrolysis (possibly PHS1)	[58]		Facilitated starch hydrolysis, making ethanol production more efficient and therefore less expensive [58].

^a^ Part of a private sector collaboration for variety development to benefit growers and processors. ^b^ One of about 24 genebank accessions from nine countries that appear to be duplicates based on molecular analysis [16]. ^c^ Pers, comm. C. Thuy.

**Table 10:** Adoption of improved cassava varieties in different Asian countries.

Country	% Adoption	CIAT Contributions Through Improved Germplasm
Cambodia	100.0	High ^a^
Myanmar	100.0	High ^b^
Thailand	99.3	High
China	98.6	Moderate
Vietnam	94.8	High ^b^
India	68.7	Low
Lao PDR	56.9	High ^a^
Indonesia	47.4	Moderate
The Philippines	44.9	Moderate
Asia	82.7	

Sources: for % adoption—Ref. [93]; for CIAT contributions—unpublished CIAT cassava program data and Table S9. ^a^ Including indirect contributions through Thailand and Vietnam. ^b^ Including indirect contributions through Thailand.

**Table 11:** Top ten cassava varieties used in South and Southeast Asia, 2017 ^a^.

Variety	Area Planted (ha)	Main Countries Where Grown
KU 50	1,302,373	Thailand, Vietnam, Cambodia, Lao PDR, Myanmar, the Philippines
Rayong 5	387,114	Thailand, Myanmar, Lao PDR, Cambodia, the Philippines
UJ 3	257,846	Indonesia
SC 205	175,846	China
UJ 5	163,537	Indonesia
Rayong 90	126,997	Thailand
Rayong 60	93,118	Thailand, Vietnam, Cambodia, Myanmar
KM 140	88,688	Vietnam
Rayong 72	83,361	Thailand, Vietnam, Lao PDR, Cambodia
UPLCa-2	63,825	The Philippines

Source: Ref. [93]. ^a^ We note that many of these data are from personal interview sources and require future verification through designed impact studies.

**Table 12:** Area and yield in Southeast Asia’s five largest cassava-producing countries from 1972 to 2022.

	Yield (t/ha)		Increase		Area (ha)		Increase	
	1972	2022	t/ha	%	1972	2022	ha	%
Cambodia	11.0	23.3	12.3	112	1900	758,607	756,707	39,827
China	n/a	16.6	n/a	n/a	180,792	304,568	123,776	68
Indonesia	7.1	27.2	20.1	283	1,468,412	499,104	−969,308	−66
Thailand	12.1	21.5	9.4	78	327,680	1,587,369	1,259,689	384
Vietnam	7.2	20.1	12.9	179	142,100	528,891	386,791	272
Total					2,120,884	3,678,539	1,557,655	73.4

Source: FAOSTAT.

**Table 13:** Comparative features of cassava landraces and elite lines related to their potential contribution to future heterotic group/pool development.

Feature	Landraces	Elite Lines
Genetic diversity	Very high	Moderate
Adaptation traits	Broad, local	Targeted
Genetic load	High	Reduced
Predictability	Low	High
Immediate hybrid value	Moderate	High
Long-term heterotic potential	Very high	Moderate

## Data Availability

Data used to develop this paper were derived from a broad range of CIAT internal institutional documents, along with public access databases Cassavabase.org and genesys-pgr.org/wiews/COL003.

## Supplementary Materials

The following supporting information can be downloaded at: https://www.mdpi.com/article/10.3390/plants15162462/s1, Table S1: Complete listing of CIAT Manihot accessions with Genesys DOI and link to evaluation and crossing information on Cassavabase.org. Table S2: Initial selections from cassava germplasm collection of about 2000 accessions in 1973–1974, based mainly on yield and harvest index performance at CIAT headquarters station. Table S3: Selected root quality traits from genebank accession evaluations during 1999–2003. Table S4: Elite hybrids identified from the CIAT Breeding Program, 1983 to 1998. Most most of them incorporated into the genebank. Table S5: Key genebank accessions selected as the most frequently appearing hybrids for adaptation to different edapho-climatic zones. Table S6: Pedigrees of over 40,000 crosses registered on Cassavabase.org by CIAT’s cassava breeding group and its partners up to 2026, illustrating the broad use of genebank accessions. Table S7: Genebank accessions in the pedigrees of CIAT’s cassava crossing nursery parents (2021–2025) across priority pipelines and trait development/deployment populations. Table S8: Reported sources of resistance in CIAT’s cassava germplasm collection to cassava brown streak disease and cassava mosaic disease, as well as tentative duplicate accessions within SNP groups. Table S9: Released varieties by region and country, based partially or fully on CIAT’s cassava genebank accessions in the genealogy.
