# Supplementary material for: Adding Value to Cassava Genetic Resources Conserved at CIAT—Part II: Fifty Years of Evaluation and Use of Landraces for Variety Development
Source: Plants (Basel). 2026 Aug 13;15(16):2462. doi: 10.3390/plants15162462 (PMC13516462; doi:10.3390/plants15162462)
Supplement: Supplementary file 1 [file plants-15-02462-s001.zip › Table S3. Quality evaluations.pdf]

Supplementary Table S3. Selected root quality traits from genebank accession evaluations 1999–2003.

Origin	No. of acces-sions	Dry matter (%)	Total sugar (%)	Amylose content (%)	Cyanide content (ppm)	Paste clarity	Pasting temp. (C)	Peak visco-sity	Break-down visco-sity	Ease of cooking (1=difficult 5=easy)
ARG	42	32	4.2	21	265	40	67	721	242	3.0
BRA	912	33	3.5	21	507	44	65	767	299	2.7
COL	1198	33	3.9	21	303	45	65	781	307	2.7
CR	86	31	3.8	21	401	49	66	705	275	2.6
CUB	62	33	4.1	21	232	44	66	784	271	3.0
ECU	82	33	3.5	21	156	48	64	855	365	2.6
GUA	59	32	3.6	21	221	45	66	816	332	2.5
IND	31	33	3.9	21	490	47	66	754	275	2.8
MAL	44	32	3.5	21	247	41	66	774	264	3.0
MEX	66	33	3.8	21	219	42	66	787	278	3.0
PAN	29	35	2.9	21	150	43	66	721	244	2.9
PAR	94	34	3.8	21	198	45	66	728	263	2.9
PER	78	33	3.2	21	163	44	65	828	322	2.8
VEN	158	33	3.9	21	310	45	65	785	295	3.0
Total[a]	2941									
Overall mean		33	3.7	21	341	44	65	777	301	2.8
Minimum		14	0.2	15	14	13	59	152	35	1.1
Maximum		48	18.8	26	3274	86	71	1402	859	5.0
Total	3221									

[a]Individual country means excluded for countries with fewer than 20 accessions evaluated, excepting in summary statistics.

Source: Genesys-pgr.org
