# Supplementary material for: Adding Value to Cassava Genetic Resources Conserved at CIAT—Part II: Fifty Years of Evaluation and Use of Landraces for Variety Development
Source: Plants (Basel). 2026 Aug 13;15(16):2462. doi: 10.3390/plants15162462 (PMC13516462; doi:10.3390/plants15162462)
Supplement: Supplementary file 1 [file plants-15-02462-s001.zip › Table S8 Sources of resistance CMD CBSD.pdf]

Supplementary Table S8. Reported sources of resistance in the CIAT cassava germplasm collection to cassava brown streak disease and cassava mosaic disease, and tentative duplicate accessions within SNP groups.

	Sources of resistance to cassava brown streak disease (CBSD) [50]							Sources of resistance to cassava mosaic disease (CMD) [52]		
Reported as resistant:	COL40	COL144	COL2182	ECU41	PER315	PER353	PER556	CR63, TAI9	PER262	VNM8
SNP Group: [16]	2998	3544	3517	2889	3188	4325	5081	127	2757	548
Other accessions in group:	COL40A	COL159	COL2186	BRA36	PER260	(None)	PER437	BRA228	(None)	(None)
			COL2108	COL1948	PER269		PER455	BRA235		
				ECU22				BRA237		
				ECU25				BRA790		
				ECU26				BRA996		
				ECU27A				BRA1050		
				ECU29				BRA1232		
				ECU36				BRA1322		
				ECU41				BRA1418		
				ECU43				COL200		
				ECU47				COL1933		
				ECU50				CR72		
				ECU138				CR106		
				ECU140				CR135		
				ECU142				CUB64		
				ECU143				CUB73		
				ECU145				ECU200		
				ECU147				FJI5		
				ECU155				GUA81		
				PAN38				IND75		
				PAN39				IND76		
				PAN39A				IND94		

Supplementary Table S8. Reported sources of resistance in the CIAT cassava germplasm collection to cassava brown streak disease and cassava mosaic disease, and tentative duplicate accessions within SNP groups.

	Sources of resistance to cassava brown streak disease (CBSD) [50]							Sources of resistance to cassava mosaic disease (CMD) [52]		
Reported as resistant:	COL40	COL144	COL2182	ECU41	PER315	PER353	PER556	CR63, TAI9	PER262	VNM8
SNP Group: [16]	2998	3544	3517	2889	3188	4325	5081	127	2757	548
				VEN16				IND96		
				VEN44				IND103		
								IND108		
								IND115		
								IND117		
								IND120		
								IND131		
								IND168		
								IND180		
								IND215		
								IND252		
								PER181		
								PER378		
								PER429		
								SLV5		
								SLV7		
								SLV8		
								VEN31		

Total countries as sources of resistance:

CBSD: Brazil, Colombia, Ecuador, Panama, Peru, Venezuela

CMD: Brazil, Colombia, Costa Rica, Cuba, Ecuador, Fiji, Guatemala, Indonesia, Peru, El Salvador, Venezuela, Vietnam
